# Label‐Free and Microplate‐Based Dissection of Glycan‐Virus Interactions Using Polymer‐Tethered Glyconanoparticles

**DOI:** 10.1002/smtd.202500214

**Published:** 2025-07-02

**Authors:** Sarah‐Jane Richards, Simona Chessa, Lloyd Sayer, Irina Ivanova, Sanaz Ahmadipour, Alexander N. Baker, Marc Walker, Simone Dedola, Katherine A. Scott, Oliver Dibben, Robert A. Field, Matthew I. Gibson

**Affiliations:** ^1^ Department of Chemistry University of Warwick Coventry CV4 7AL UK; ^2^ Warwick Medical School University of Warwick Coventry CV4 7AL UK; ^3^ Department of Physics University of Warwick Coventry CV4 7AL UK; ^4^ Department of Chemistry University of Manchester Oxford Road Manchester M13 9PL UK; ^5^ Manchester Institute of Biotechnology University of Manchester 131 Princess Street Manchester M1 7DN UK; ^6^ Iceni Glycoscience Norwich Research Park Norwich NR4 7TJ UK; ^7^ Flu‐BPD Biopharmaceuticals R&D AstraZeneca Renaissance Way Speke Liverpool L24 9JW UK

**Keywords:** biosensing, glycans, influenza, nanoparticles, polymers, sialic Acid

## Abstract

Influenza viruses use haemagglutinins (HA) to target host sialic acids in the respiratory tract as do other pathogens, including coronaviruses, which engage using spike protein. The host adaptation of the HA protein, which leads to the accumulation of mutations, is a key descriptor of individual influenza strains, which aids zoonosis and is crucial in vaccine development. How each strain targets glycans is crucial to understanding function, designing new therapies, and optimizing candidates for vaccine development. Here, it is demonstrated that polymer‐tethered plasmonic (gold) glyconanoparticles can be deployed for rapid evaluation of whole influenza virus binding, readable by simple UV–vis within a microwell plate as a low‐tech alternative to printed microarrays. It is also demonstrated that the synthetic methodology is compatible with large branched glycans from chemoenzymatic synthesis, allowing a wider range of glycan structures to be probed. Particles are obtained by a modular capture and immobilisation process and used to interrogate the binding of five influenza strains as proof of concept. These results show that glycosylated nanoparticle probes are suitable for the rapid interrogation of live virus to map how glycan structure impacts binding and can enable at‐bench, rapid virus/glycan binding readouts and aid the development of interventions for influenza and other viruses.

## Graphical abstract

This work demonstrates the use of polymer‐tethered glycosylated gold nanoparticles for the label‐free evaluation of influenza virus binding in a simple microplate. Sialylated linear and branched glycans are integrated using a modular capture and assembly process. The microplate‐based assays are shown to correlate with biolayer interferometry and can be conducted at the benchside for rapid evaluation.

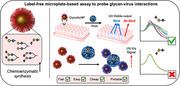

## 1 Introduction

Carbohydrates and their binding proteins (lectins) play wide‐ranging roles in biological homeostasis.^[^ ^1^ ^]^ They also underpin host‐pathogen interactions during the infection process, with terminal sugar sialic acid (neuraminic acid) a dominant player in such recognition events.^[^ ^2^ ^]^ The significance of sialic acid binding in relation to influenza virus infection, in particular, has been extensively studied,^[^ ^3^ ^]^ both in relation to human health and also zoonotic infection (avian, swine).^[^ ^2^ ^]^ In addition to influenza, a wide range of other respiratory viruses engage cell surface sialic acids,^[^ ^4^ ^]^ including coronaviruses^[^ ^5^, ^6^, ^7^, ^8^, ^9^ ^]^ (SARS‐CoV‐2). More broadly, other pathogens also engage a diverse range of cell surface glycan targets,^[^ ^10^ ^]^ and hence deciphering these interactions is highly important in the context of developing diagnostic devices for potential zoonotic threats, therapeutic agents, and live attenuated vaccines.

Influenza viruses interact with sialic acid on the host through their haemagglutinin (HA) proteins, while the neuraminidase (NA) protein is responsible for sialic acid cleavage during the release of replicated virus.^[^ ^3^ ^]^ HA binds to the terminal sialic acid unit and also to part of the underlying glycan to which it is attached. Hence, influenza viruses are able to discriminate between prospective hosts through binding with specific sialylated oligosaccharide structures.^[^ ^11^, ^12^ ^]^ In human upper respiratory tract tissues where the predominant glycans contain α‐2,6‐linked sialic acid, the cognate human influenza virus HA shows α‐2,6 specificity; in contrast, avian influenza viruses bind preferentially to α‐2,3‐linked sialic acids, which are prevalent in bird respiratory and gut tissue. Pigs, on the other hand, express both α‐2,6‐ and α‐2,3‐linked sialic acid, and can be infected with both human and avian influenza viruses,^[^ ^13^ ^]^ providing a breeding ground for virus reassortment and the generation of new and potentially dangerous pathogenic strains. HA specificity can also be impacted by the length of the underlying glycan that presents sialic acid, with recent pandemic H1N1 influenza viruses showing a strong preference for a minimum di‐LacNAc structural motif.^[^ ^14^ ^]^ In addition, glycan valency has an impact on binding to the trimeric HAs of influenza A virus,^[^ ^15^ ^]^ which can be exploited in the development of efficient HA binders that block infection.

Taken together, the above information highlights the opportunity and need to develop new tools to enable rapid interrogation of glycan/pathogen interactions, whilst optimising the multivalent display of ligands. Microarray platforms are ideal for high‐throughput screening where 100′s of distinct glycans are used, but are less suited for smaller sets of ligands, requiring array printing/reading instrumentation and the use of labeled, rather than native, proteins.^[^ ^16^, ^17^ ^]^ Glycans have been integrated into phage display for high‐throughput selection and liquid phase arrays, but these are non‐trivial to use.^[^ ^18^ ^]^ The multivalent display of glycans in nanoparticle format leads to non‐linear enhancements in binding avidity due to the cluster glycoside effect^[^ ^19^, ^20^, ^21^ ^]^ and has been deployed in a range of non‐metallic materials including polymers,^[^ ^22^ ^]^ dendrimers,^[^ ^23^ ^]^ oliognucleotides^[^ ^24^ ^]^ and liposomes. Glycosylated nanomaterials have been widely deployed to inhibit pathogen binding events.^[^ ^25^ ^]^ Mannoside functional gold nanoparticles, fullerenes, and dendrimers have been shown to be capable of inhibiting Ebola virus entry into cells via DC‐SIGN blocking.^[^ ^26^, ^27^, ^28^ ^]^ Sialic acid functional nanoparticles and polymers have been widely explored for the inhibition of influenza infection.^[^ ^29^, ^30^ ^]^

However, the above examples were not used to generate a signal to allow dissection/interrogation of the binding events and require coupling to secondary assays or biophysical techniques such as SPR (surface plasmon resonance), BLI (biolayer interferometry), impedimetric sensing^[^ ^31^ ^]^ or calorimetry, as selected examples. Metal nanoparticles are a promising sensing platform, making use of their unique plasmonic properties for assays based on aggregation,^[^ ^32^ ^]^ fluorescence quenching^[^ ^33^ ^]^ or lateral flow.^[^ ^34^, ^35^ ^]^ Glycosylated polymer‐coated gold nanoparticles provide a versatile platform for the interrogation of multivalent‐multivalent interactions between glycans and lectins: the multiple binding sites lead to aggregation and, due to the surface plasmon resonance (SPR) bands, coupling a corresponding change in colour from red to blue, allowing a label‐free read‐out of binding. The polymer coating is crucial as direct immobilisation of glycans onto gold particles may not give colloidal stability (due to a lack of steric stabilisation) in complex (saline) media, leading to false‐positives, but conversely, the length of the polymer must be tuned to prevent over‐stabilisation and false‐negatives.^[^ ^36^, ^37^ ^]^ Field and Russell used PEG‐linked glyconanoparticles to probe glycan interactions with cholera toxin and with influenza virus strains.^[^ ^38^, ^39^, ^40^ ^]^ Richards et al. have used glycosylated particles to probe HA protein, coronavirus spike protein, and galectin binding to glyconanoparticles using a poly(2‐hydroxyethyl acrylamide) tether.^[^ ^6^, ^41^, ^42^ ^]^ An advantage of the glyconanoparticle system is that it is easy to deploy with minimal infrastructure, which may be of use for surveillance or rapid response to emerging pathogens, their variants, and vaccine strain selection. However, many studies rely on purified proteins rather than viruses and use glycoparticles with limited glycan complexity, relying on (typically) trisaccharides or smaller. These do not fully capture the complex multivalent‐multivalent interactions that give rise to the overall selectivity and affinity of a native system.

The aim of this work was to deploy the polymer‐tethered glyconanoparticle label‐free biosensing platform to enable the capture of large and increasingly complex biologically‐relevant glycans, and to use these to evaluate the binding of intact virus without the need for separate protein expression or isolation steps. Here, a panel of sialylated mono‐ and bi‐antennary glycan mimetics is prepared by a chemoenzymatic strategy and coupled to polymer tethers for successful immobilisation onto gold nanoparticles. The resulting glyconanoparticles are then used to interrogate a panel of live attenuated influenza vaccine (LAIV) virus strains. LAIV A viruses are uniform across most of their genome, with only HA/NA varying, making them a convenient model. It is found that the microplate‐based colourimetric screening aligns very well with a (more complex) biolayer interferometry‐based binding assay, but it is faster and easier to use. Crucially, we are not intending this to be used as a diagnostic, but this platform is suitable for the rapid evaluation of glycan binding using intact virus, to understand host‐pathogen interactions, aid the discovery of glycomimetics, develop new tools for pathogen surveillance, and screening suitable vaccine candidates.

## 2 Results and Discussion

**Figure 1:**
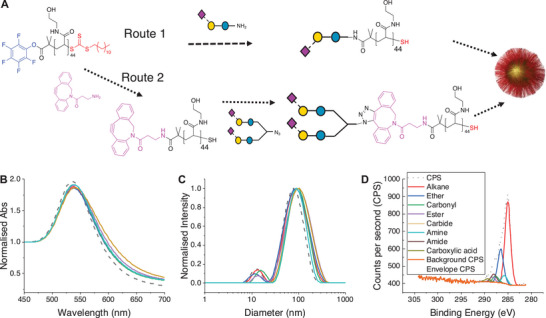
Modular synthetic approach to obtain gold nanoparticles functionalised with complex glycans. A) Synthesis of glycosylated gold nanoparticles via a 1‐ or 2‐step polymer end‐group conjugation; B) UV–vis spectra of AuNP panel; C) Dynamic light scattering size distribution of the AuNP panel; D) Representative XPS spectra of 2,3SL‐PHEA_44_@AuNP_55_.

**Figure 2:**
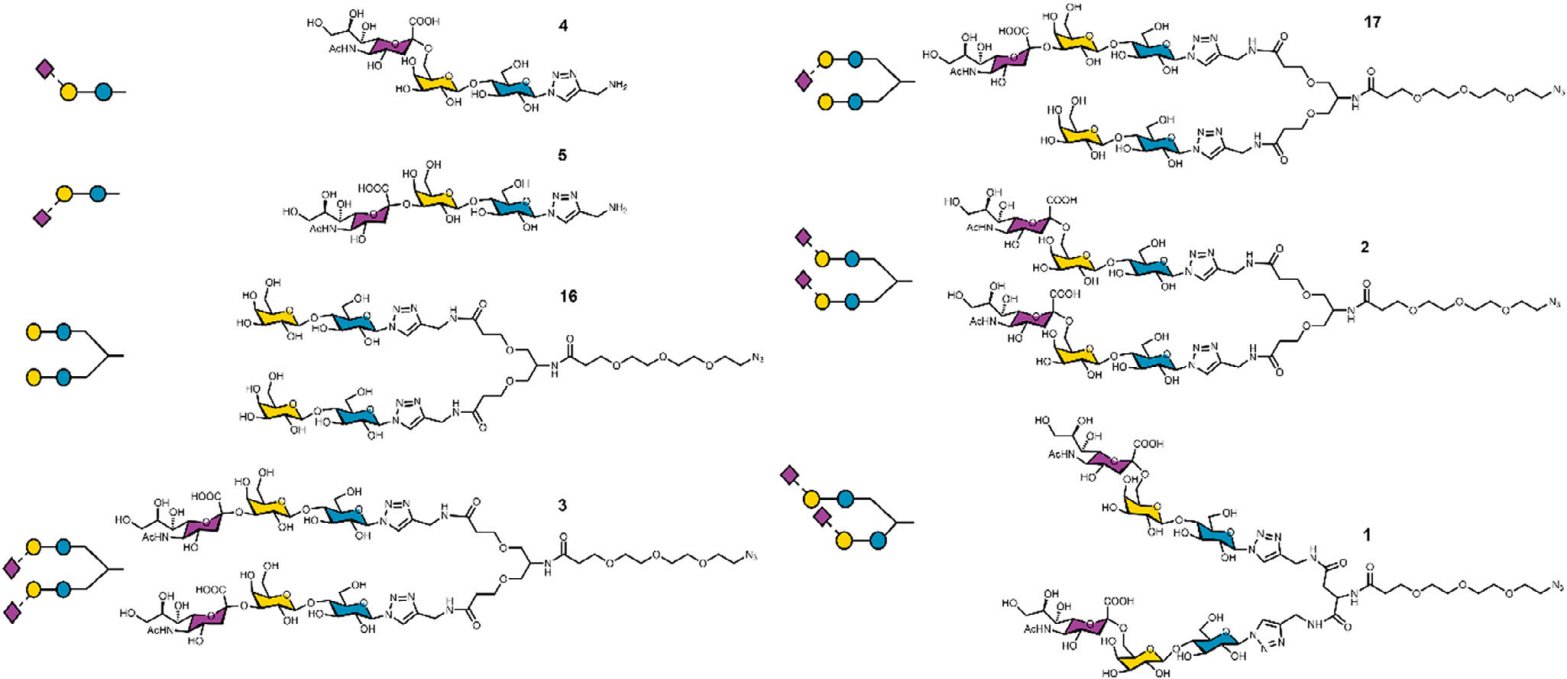
Glycans used in this study, showing chemical structure and symbol nomenclature for each glycan. See supporting information for chemoenzymatic synthesis and characterisation of each material.

Our design strategy involves the capture of mono‐ and bi‐antennary sialylated glycan mimetics onto polymeric tethers, which are then immobilised onto pre‐formed gold nanoparticles. The plasmonic gold particles are the primary components of the UV–vis/microplate based biosensor (**Figure** **1**). This approach ensures colloidal stability (due to steric stabilisation by the polymers), the gold particle immobilisation leads to multivalent display, and the gold itself provides a handle for spectroscopic analysis of binding (Figure 1). The polymeric tether was based on the pentafluorophenyl ester‐terminated poly(*N*‐hydroxyethyl acrylamide) (PFP‐PHEA_44_) platform obtained by RAFT (reversible addition fragmentation transfer) polymerisation.^[^ ^6^, ^41^, ^43^ ^]^ This polymer is both water soluble and resistant to hydrolysis (unlike, e.g., acrylate/methacrylates), making it suitable for further modification. The PFP handle can be displaced by either amino‐glycans (Figure 1A, route 1), or in two steps by introduction of a SPAAC (strain promoted azide‐alkyne cycloaddition (Figure 1, route 2) click unit, to capture azido‐glycans, and then immobilised onto 55 nm gold nanoparticles (other diameters can be used but this size was suitable for the viral probes used in this study). The azido route was found to be more effective when using very low (<1 mg) quantities of the glycans. Azido glycans do not need an extra step to reduce to amine, and SPAAC is carried out in water therefore can be used without purification for AuNP immobilisation, as excess glycan is removed by centrifugation/resuspension cycles. The excess, unbound polymer is also removed through centrifugation/resuspension cycles. A panel of glycans was synthesised (**Figure** **2**) and used to assemble a library of seven types of glyconanoparticles, each one bearing a different mono/bi‐antennary glycans. The nanoparticles were characterised by UV–vis spectroscopy (Figure 1B) and dynamic light scattering (Figure 1C), confirming their size and colloidal stability. X‐ray photo‐electron spectroscopy (XPS) was used to confirm the presence of the polymers/glycans on the nanoparticle surface. XPS is ideally suited to this, as it requires only very low quantities of material, unlike, e.g., NMR approaches or TGA To give an indication of grafting density, related systems can give ≈0.3 chains.nm^−2^ providing sufficient glycans for multivalent enhancement and to cross‐link with the virus.^[^ ^44^ ^]^

A focused panel of mono and bi‐antennary sialylated glycan mimetics was prepared to enable exploration of this bio‐sensing approach, to be used against a panel of influenza viruses. The synthetic methods result in either amino or azido functionality for polymer‐conjugation, chosen for both synthetic accessibility and to probe the benefits of the polymer‐tether, which can be easily adapted to several conjugation strategies. The glycan mimetics were designed with ease of chemoenzymatic synthesis in mind, while emulating the overall distances between biantennary core and terminal sialic acid found in native sialylated *N*‐glycans (Figure S1, Supporting Information). Where possible, commercially available 2,3 and 2,6 sialyllactose were used as building blocks. The enzymatic installation of sialic acid at the 6‐OH or 3‐OH of terminal galactose residue(s) was obtained, respectively, using commercial recombinant α‐2,6‐sialyltransferase from *Photobacterium damsel* (Pd26ST) and *Trypanosoma cruzi trans‐sialidase* (TcTS).^[^ ^45^, ^46^ ^]^ All final compounds were characterised with a combination of 1D and 2D NMR and ESI‐MS or MALDI‐ToF mass spectrometry. It should be noted that these glycans were chosen to develop the analytical tools, rather than being the precise physiologically relevant glycans from hosts.

Initial screening (not shown) confirmed that the glyconanoparticles presented the appropriate diameter (55 nm) and architecture to give colourimetric readouts and were colloidally stable in the assay conditions, preventing false positives. Furthermore, control nanoparticles (no glycan) did not aggregate in response to the viruses. This is essential as we have previously demonstrated how particle diameter, polymer tether length, and chemistry can be used to tune the binding interactions to lectins, and that incorrect composition can lead to false positives or negatives against glycan binding proteins.^[^ ^37^, ^43^ ^]^ With the library to hand, our primary aim was to interrogate in a more native environment, hence the use of intact virus rather than HA protein alone,^[^ ^42^ ^]^ as the density of the HA surface proteins can impact binding outcomes. All assays were undertaken in the presence of neuraminidase inhibitors (oseltamivir carboxylate and zanamivir), ensuring results were not biased through neuraminidase cleavage of sialic acids, as demonstrated by Benton et al.^[^ ^47^ ^]^

**Figure 3:**
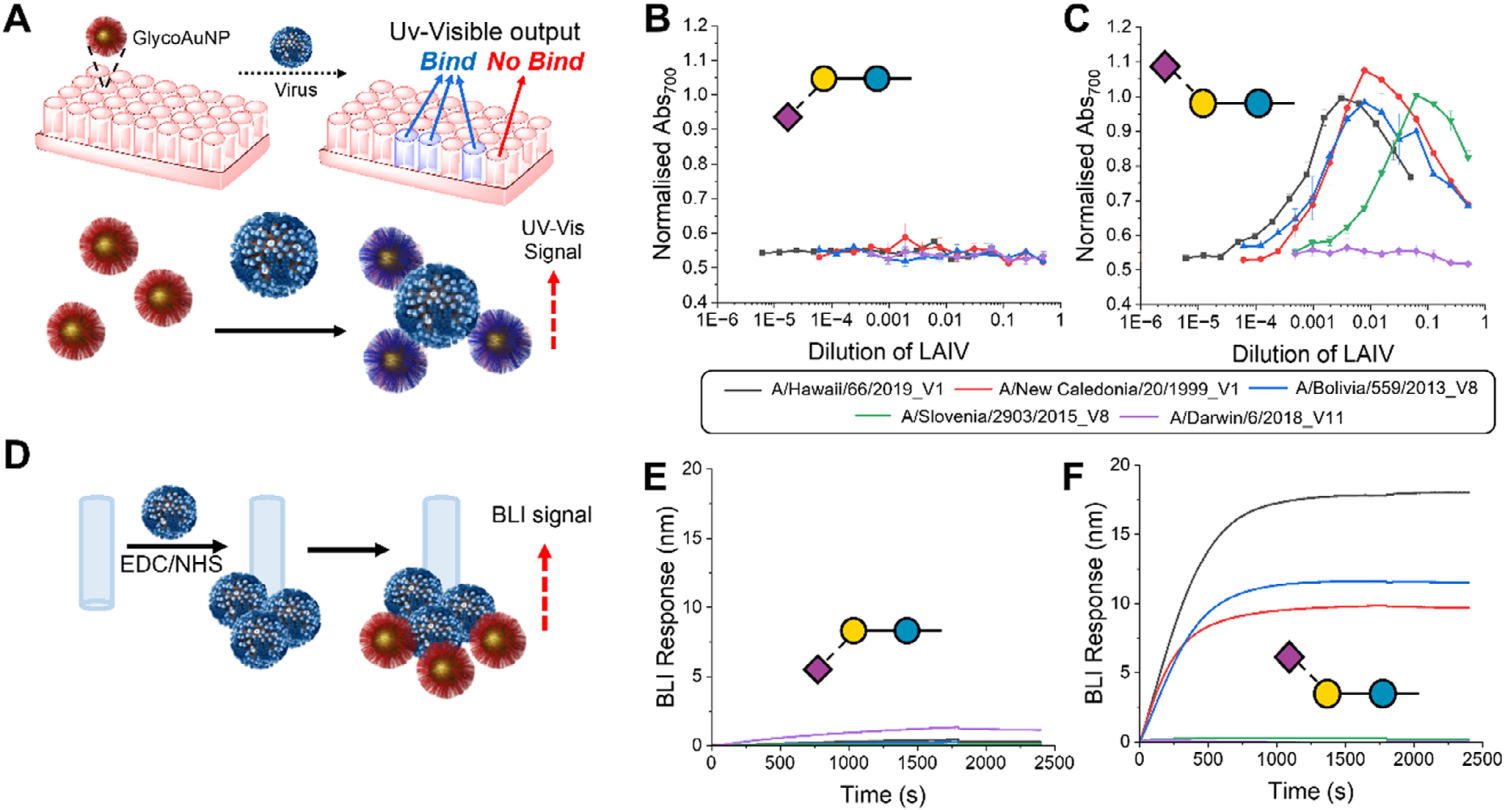
Sialyllactose‐functional nanoparticles binding to the LAIV virus. A) Schematic representation of the aggregation assay, where GlycoAuNP particles cross‐linking virus; B) Dose response of LAIV viruses versus 2,3 sialyllactose AuNPs; C) Dose response of LAIV viruses versus 2,6 sialyllactose AuNPs; Error bars are the standard error of the mean from n = 3. D) Schematic representation of the BLI assay with immobilisation of the LAIV virus to the sensor; E) BLI response of LAIV virus to 2,3 SL AuNPs; F) BLI response of LAIV virus versus 2,6 SL AuNPs. BLI was undertaken at 0.375× dilution of LAIV virus with AuNPs at OD_540_ (optical density @ 540 nm) = 1. In all assays, neuraminidase inhibitors, 25 µM oseltamivir carboxylate and 100 µM zanamivir, were used.

The first glycans tested were the monoantennary 2,3 and 2,6 sialyllactose derivatives (**Figure** **3**). It is well established that human influenza viruses have a preference for 2,6‐linked sialic acids, while avian influenza (a source of zoonotic influenza viruses) has a preference for the 2,3‐linked equivalent.^[^ ^12^, ^39^ ^]^ To evaluate binding, a panel of five H1N1 LAIV strains carrying HA/NA genes isolated from between 1999 and 2019 were incubated with the glycosylated nanoparticles, which due to the multivalent‐multivalent interactions leads to aggregation and a clear red‐blue colour shift due to coupling of the AuNPs SPR bands (Figure 3A). Figure 3B,C shows the response of the glycan functionalised (2,3 sialyllactose (2,3 SL) and 2,6 sialyllactose, respectively) AuNPs to the LAIV by the change in the absorbance at 700 nm. The full curves for these are shown in ESI (Figures S11–S17, Supporting Information). The 2,3 SL glycan shows little to no response to the LAIV viruses (Figure 3B) but in the case of the 2,6 sialyllactose (2,6 SL) (Figure 3C), there was a significant change in the absorbance. A/Hawaii/66/2019_V1 exhibits the strongest binding to 2,6 SL. A/New Caledonia/20/1999_V1 and A/Bolivia/559/2013_V8 bind to the same extent by this aggregation method, and A/Slovenia/2903/2015_V8 binds but exhibits 10× lower values. In this assay, A/Darwin/6/2018_V11 shows no binding to 2,6 SL across the virus concentration range used in this work, suggesting that longer and more complex linkers (such as polyLacNAc^14^) are required for effective binding or the virus concentrations are beyond the limits of this assay format.

To further validate the results, a complementary biolayer interferometry‐based (BLI) assay was also used (Figure 3D). In this case, LAIV viruses were covalently immobilised onto amine reactive BLI sensors (ARG2) using EDC/NHS coupling (Figures S1–S3, Supporting Information). The glyconanoparticles could then be exposed, and the BLI signal measured (Figure 3E,F). The dataset obtained with the two different assays (aggregation and BLI) correlate very well, suggesting that A/Hawaii/66/2019_V1 binds to 2,6 SL to the greatest extent, followed by A/Bolivia/559/2018_V8 and A/New Caledonia/20/1999_V1 with Bolivia giving slightly higher response to New Caledonia which is not discerned in the aggregation assay. Only a weak response is observed with A/Slovenia/2903/2015_V8, and no response is observed with A/Darwin/6/2018_V11, which mirrors the results obtained with the aggregation assay (Figure 3F). At high viral concentrations, the hook effect is observed, whereby the signal begins to decrease due to saturation of the particle surface. This is a key consideration for this aggregation method and highlights why it is important to survey a concentration range, which is very easy and low cost with this method, which is not the case for glycan arrays or SPR technologies. No, or very little, binding is shown to 2,3 SL (Figure 3E). Interestingly, here, a very small response is seen to A/Darwin/6/2018_V11. It is very encouraging that the two assays correlate well and the BLI backs up the easier, lower‐resource aggregation method. The aggregation assay is rapid, high‐throughput, and only requires a plate reader, moreover, the response can also be seen by eye, due to the colour change from red to blue therefore is easily deployable to use in any laboratory or other environment.

**Figure 4:**
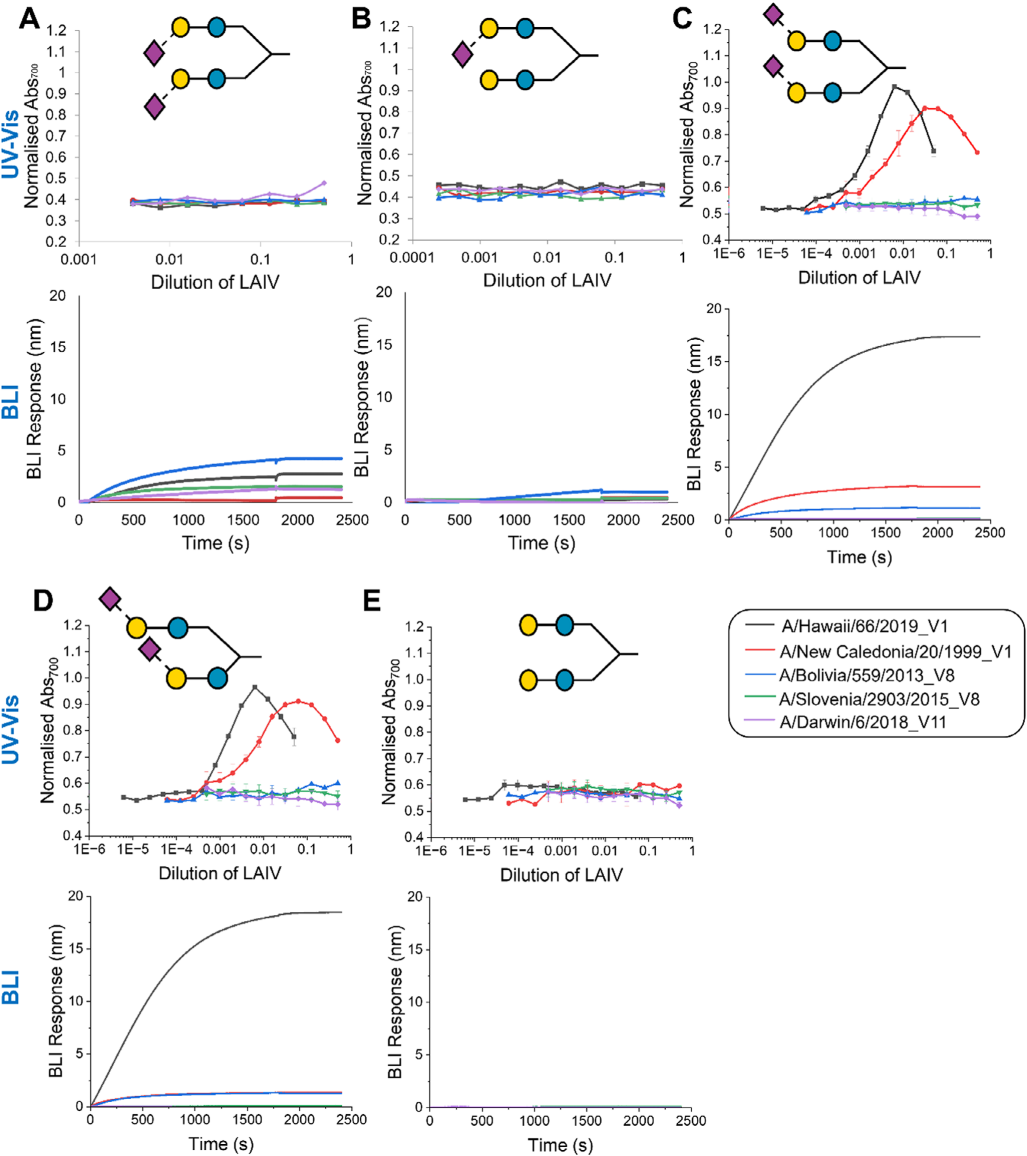
Comparison of UV–vis spectroscopy and biolayer interferometry (BLI) binding of intact LAIV virus to glyconanoparticles. For each subsection, top: dose response of LAIV virus versus biantennary glycosylated‐AuNPs by UV–vis and bottom: BLI response of LAIV virus to biantennary glycan‐AuNPs where the glycan is: A) biantennary 2,3 SL (**3**), B) mono‐functionalised biantennary 2,3 SL (**17**), C) biantennary 2,6 SL (**2**), Error bars are the standard error of the mean from n = 3. D) Asymmetric biantennary 2,6 SL (**1**), E) biantennary lactose (**16**). BLI was undertaken at 0.375× dilution of LAIV virus with OD 1 AuNPs. In all assays, neuraminidase inhibitors, 25 µM oseltamivir carboxylate and 100 µM zanamivir, were used.

With proof of whole LAIV virus binding shown using the trisaccharides, the more complex branched‐glycans were then interrogated using both assays, with the UV–vis (which can be conducted in 96 well plates) suitable to obtain full dose response and the BLI for a fixed concentration, **Figure** **4**. As observed for the simple disaccharides, there was strong agreement between the BLI and the microplate assay, reconfirming that this approach can be deployed to screen glycans with more complex structures. A negative control of biantennary lactose (Figure 4E) was included, showing no binding to any strains by either method: confirming that the binding is driven by the glycan structure and there is no non‐specific binding to the polymer‐coated AuNPs, which is crucial in all biosensors. Both bi‐ (Figure 4A) and mono‐ (Figure 4B) 2,3 SL terminal glycans showed limited absorbance shift in response to the LAIV viral panel, with small changes at the highest concentrations of virus. The biantennary 2,3 SL did, however, show a small response in the BLI. We attribute this to the higher sensitivity of the BLI method, able to detect even very weak off‐specific interactions. Biantennary symmetric 2,6 SL (Figure 4C) was identical to a non‐symmetric biantennary 2,6 SL (Figure 4D) by UV–vis, whereas the BLI does not show increased binding to the A/New Caledonia/20/1999_V1. Furthermore, there is a small change at the highest concentration tested for A/Darwin/6/2018_V11 observed by UV–vis for the symmetric version (Figure S15E, Supporting Information) that is not seen for the non‐symmetric (Figure S16E, Supporting Information) for reasons that are unclear. Interestingly, A/Bolivia/559/2013_V8 did not show binding to the biantennary 2,6 SLs, but binding was observed to the mono‐2,6 SL, whereas A/Hawaii/66/2019_V1 and A/New Caledonia/20/1999_V1 were found to be capable of binding to both the symmetric and non‐symmetric biantennary 2,6 SLs. These data fully validate the combined synthetic and analytical approaches to using polymer‐tethered glyconanoparticles for the rapid dissection of intact viral binding interactions, to screen for new function. This has many advantages, including allowing rapid interrogation of viral samples in containment laboratories where the simple read‐out can be used and may aid in future pandemic or outbreak surveillance and vaccine strain selection. For example, during COVID‐19, glycans emerged as key players,^[^ ^6^, ^7^ ^]^ but faster and more adaptable tools for their deployment are required.

## 3 Conclusion

Herein, the capture of complex mono‐ and bi‐antennary sialic‐acid terminated glycan mimetics onto polymer tethered glyconanoparticles is demonstrated, and their subsequent use for label‐free interrogation of glycan‐virus binding interactions as an alternative to printed microarrays. Glycans were assembled using chemoenzymatic synthesis to allow installation of the sialic acids in either 2,3 or 2,6 regio‐isomers to give simplified structural motifs associated with avian and human influenza hemagglutinin binding. These glycans were immobilised via amine/pentafluorophenyl, or azide/dicyclobenzyloctyne conjugations onto poly(2‐hydroxyethyl acrylamide), equipped with a terminal thiol for assembly onto pre‐formed gold nanoparticles. The gold nanoparticles provide multivalent display and signal enhancement in the subsequent binding assays. These particles were used to interrogate five LAIV candidate vaccine viruses (CVVs) as model systems to demonstrate that the technology can be adapted from previous reports of protein binding to intact viral particles. It was shown that a simple microplate‐based assay, with a red‐blue colour change due to surface plasmonic resonance coupling of the gold particles, could be used to determine the relative affinity of the different glycan/virus combinations using UV–vis spectroscopy. This approach removes the need for any secondary detection agents, in contrast to typical microarray‐based assays. The binding results were further validated using biolayer interferometry (which is also label‐free), confirming the binding observations from UV–vis. From the results of both assays, the 2,3‐linked sialic acids and control lactose (no sialic acid) glycans showed significantly less binding than 2,6 linked sialic acids. It is important to note that these viruses were egg‐adapted and the glycans used here are not representative of the chorioallantoic membrane of eggs, and are distinct from, e.g., avian glycans which are relevant for, e.g., zoonosis. Nonetheless, this validates the technology and the workflow from chemoenzymatic synthesis to plasmonic biosensing with viral particles. Future work will focus on deploying these tools with glycans that are more representative of the human upper respiratory tract that human‐adapted influenza viruses are targeting for cell entry. Furthermore, these tools may aid to identify glycans that from the cell surface of egg chorioallantoic membranes in embryonated chicken egg propagation, which could aid in building a predictive tool for viral binding as an indicator of egg yield. In summary, these results validate that polymer‐tethered glyconanoparticles are a powerful tool for rapid, benchside interrogation of virus/glycan interactions without needing specialised facilities and in a fully label‐free manner.

## Conflict of Interest

KAS and OD are employees of AstraZeneca and may/may not hold stocks and shares.

## Supporting information



Supporting Information

## Data Availability

The data that support the findings of this study are available in the supplementary material of this article.
