# Supplementary material for: Label‐Free and Microplate‐Based Dissection of Glycan‐Virus Interactions Using Polymer‐Tethered Glyconanoparticles
Source: Small Methods. 2025 Jul 2;9(8):2500214. doi: 10.1002/smtd.202500214 (PMC12391648; doi:10.1002/smtd.202500214)
Supplement: Supplementary file 1 — Supporting Information [file SMTD-9-2500214-s001.pdf]

small
methods

Supporting Information

for *Small Methods*, DOI 10.1002/smtd.202500214

Label-Free and Microplate-Based Dissection of Glycan-Virus Interactions Using Polymer-Tethered Glyconanoparticles

*Sarah-Jane Richards, Simona Chessa, Lloyd Sayer, Irina Ivanova, Sanaz Ahmadipour, Alexander N. Baker, Marc Walker, Simone Dedola, Katherine A. Scott, Oliver Dibben, Robert A. Field and Matthew I. Gibson**

Electronic Supporting Information for

Label-Free and Microplate-Based Dissection of Glycan-Virus Interactions using Polymer-Tethered Glyconanoparticles

Sarah-Jane Richards,[a,e] Simona Chessa,[g] Lloyd Sayer,[g] Irina Ivanova,[g] Sanaz Ahmadipour,[e,f,g] Alexander N. Baker,[a] Marc Walker,[c] Simone Dedola,[g] Katherine A. Scott,[h] Oliver Dibben,[h] Robert A. Field,[e,f,g] Matthew I. Gibson[a,b,e,f]

a) Department of Chemistry, University of Warwick, Coventry, CV4 7AL, United Kingdom

b) Warwick Medical School, University of Warwick, Coventry, CV4 7AL, United Kingdom

c) Department of Physics, University of Warwick, Coventry, CV4 7AL, United Kingdom

e) Department of Chemistry, University of Manchester, Oxford Road, Manchester, M13 9PL, UK

f) Manchester Institute of Biotechnology, University of Manchester, 131 Princess Street, Manchester, M1 7DN, UK

g) Iceni Glycoscience, Norwich Research Park, Norwich, NR4 7TJ, UK

h) Flu-BPD, Biopharmaceuticals R&D, AstraZeneca, Renaissance Way, Speke, Liverpool L24 9JW, UK

CORRESPONDING AUTHOR DETAILS

Matt.gibson@manchester.ac.uk

Experimental Section

Materials and Methods

Materials

All chemicals were used as supplied unless otherwise stated. 2-(Dodecylthiocarbonothioylthio)-2-methylpropionic acid pentafluorophenyl ester (98%, PFP-DMP), *N*-hydroxyethyl acrylamide (97%, HEA), 4,4-Azobis(4-cyanovaleric acid) (≥ 98%, ACVA), triethylamine (>99%), sodium citrate tribasic dihydrate (>99%), gold(III) chloride trihydrate (99.9%), HEPES (≥99.5%) were purchased from Sigma-Aldrich. Sodium chloride (≥99.5%) and calcium chloride were purchased from Thermo Fisher Scientific. *Maackia amurensis* lectin I (MAL I) and *Sambucus nigra* (SNA) lectins were purchased from Vector Laboratories. Clear, flat bottom, half area 96-well plates and black flat bottom 96-well plates were purchased from Greiner Bio-one. Distilled water used for buffers was MilliQ grade >18.2 mΩ resistance. Amine reactive (ARG2) biosensors were purchased from Sartorius. The azido-PEG_3-NHS esters (monomer), and 2-(azido-PEG3-amido)-1,3-bis(NHS Ester) (dimer) were purchased from Broadpharm. Cytidine 5-triphosphate disodium salt (CTP), E. coli recombinantα-2,6-sialyltransferase from Photobacterium damselae (Pd26ST) and E. coli recombinant CMP-sialic acid synthetase from Neisseria meningitidis (NmCSS;) were purchased from Chemily Glycoscience. SPE tubes, Supelclean™ ENVI-Carb™, Supelco were purchased from sigma Aldrich, NAP-10 columns were purchased from GE Health care.

Characterisation Techniques

Dynamic Light Scattering. Hydrodynamic diameters (D_h) and size distributions of particles were determined by dynamic light scattering (DLS) using a Malvern Zetasizer Nano ZS with

a 4 mW He-Ne 633 nm laser module operating at 25 ºC. Measurements were carried out at an angle of 173° (back scattering), and results were analysed using Malvern DTS 7.03 software. All determinations were repeated 5 times with at least 10 measurements recorded for each run. D_h values were calculated using the Stokes-Einstein equation where particles are assumed to be spherical.

UV-Vis Spectroscopy. Absorbance measurements BioTek Epoch microplate reader in the wavelength (λ) range of 450-750 nm (step = 10 nm).

X-ray Photoelectron Spectroscopy. The X-ray photoelectron spectroscopy (XPS) data were collected at the Warwick Photoemission Facility, University of Warwick. The samples were attached to electrically-conductive carbon tape, mounted on to a sample bar and loaded in to a Kratos Axis Ultra DLD spectrometer which possesses a base pressure below 1 x 10^{-10} mbar. XPS measurements were performed in the main analysis chamber, with the sample being illuminated using a monochromated Al Kα x-ray source. The measurements were conducted at room temperature and at a take-off angle of 90° with respect to the surface parallel. The core level spectra were recorded using a pass energy of 20 eV (resolution approx. 0.4 eV), from an analysis area of 300 μm x 700 μm. The spectrometer work function and binding energy scale of the spectrometer were calibrated using the Fermi edge and $3d_{5/2}$ peak recorded from a polycrystalline Ag sample prior to the commencement of the experiments. In order to prevent surface charging the surface was flooded with a beam of low energy electrons throughout the experiment and this necessitated recalibration of the binding energy scale. To achieve this, the C-C/C-H component of the C 1*s* spectrum was referenced to 285.0 eV. The data were analysed in the CasaXPS package, using Shirley backgrounds and mixed Gaussian-Lorentzian (Voigt) lineshapes. For compositional analysis, the analyser transmission function has been determined

using clean metallic foils to determine the detection efficiency across the full binding energy range.

Thin-layer chromatography (TLC) was performed on aluminium-backed, pre-coated silica gel plates (Silica Gel 60 F254, Merck). TLC were developed by immersion in a phosphomolybdic acid stain solution, followed by heating to 200 ºC or by immersion in PPh_3 /ninhydrin specific azide staining solution Column chromatography was performed on a Biotage Isolera one™ flash chromatography purification system using pre-packed silica gel cartridges.

NMR spectra were recorded with either a Bruker Ultrashield plus 400™ spectrometer at 400 MHz (^{1}H) or 100 MHz (^{13}C) or a Bruker Ascend 800™ spectrometer at 800 MHz (^{1}H) or 200 MHz (^{13}C), in the specified deuterated solvent. ^{1}H NMR and ^{13}C NMR spectra were referenced to residual solvent peaks, for ^{13}C NMR experiment performed in D_2O methanol was added as internal reference. ^{1}H NMR spectra were assigned with the help of COSY experiments and ^{13}C NMR spectra with the help of HSQC experiments. Chemical shifts (δ) and coupling constants (*J*) are given in ppm and Hz, respectively.

In NMR assignment the following abbreviations have been used: glucose = Glc; galactose = Gal; 5-*N*-acetylneuraminic acid = NeuAc; ax = axial proton; eq = equatorial proton; triazole protons and carbons labelling is reported for each compound. NMR assignment of triazole signals and their glycosyl derivatives were supported by previous work[1] however, the ^{13}C signal of C_b and $C_{b'}$ quaternary carbons were not always detectable.

Matrix-assisted laser desorption ionisation time-of-flight mass spectrometry (MALDI-ToF-MS) analyses were performed on a Bruker Daltonics autoflex speed ToF/ToF mass spectrometer with a N_2 laser in linear 50 shots mode. Mass data were analysed using Bruker Daltonics Flexanalysis software. Samples for MALDI-ToF-MS analysis were prepared by

mixing 0.5 μL of sample at the concentration of 1 mg/mL with 0.5 μL of 2,5-dihydroxybenzoic acid (DHB) matrix at the concentration of 10 mg/mL in MeOH, 1 μL of mixture was deposited onto an MTP AnchorChip 384 target plate and allowed to air dry.

Synthetic Section

Glycan Synthesis Section

Preparation of α-2,6- (Lac-t-NH)$_2$-Asp-PEG3-N$_3$ (1)

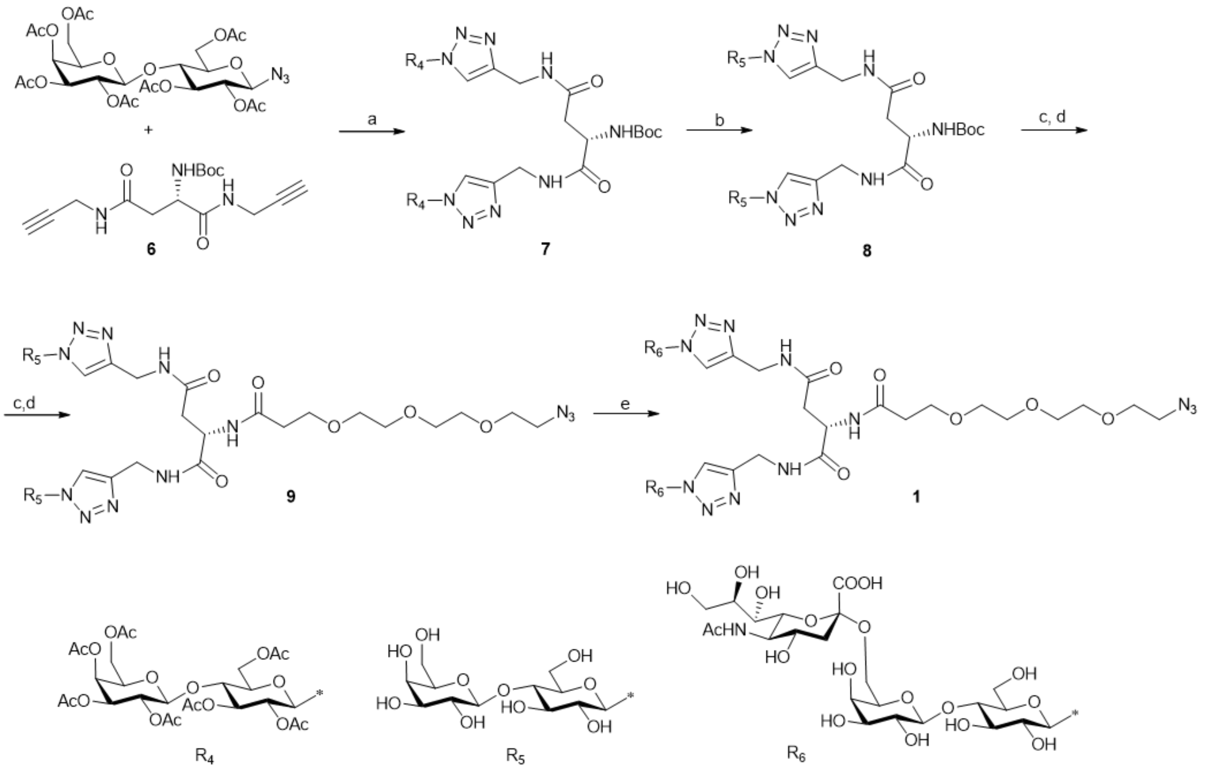

Scheme S1. Preparation of the asymmetric bi-antennary 1. Reagents and conditions: a) peracetylated lactose azide, Bis(propargylamido)-Boc-L-Asp 6, $CuSO_4$, sodium ascorbate, Tris buffer, r.t., 16 hrs; b) anhydrous MeOH, sodium methoxide (0.5 M in MeOH), r.t., 16 hrs; c) 8, anhydrous DCM, TFA, r.t., 20 minutes; d) H_2O, 1M $NaHCO_3$, azido-PEG$_3$-NHS in anhydrous DMF, Diisopropylethylamine, r.t., 3 hrs; e) 9, CTP, Neu5Ac, alkaline phosphatase, $MgCl_2$, α-2,6-SiaT, CMP-SA synthetase, Tris buffer pH 8.3, 37 °C, overnight.

Preparation of building block (6)

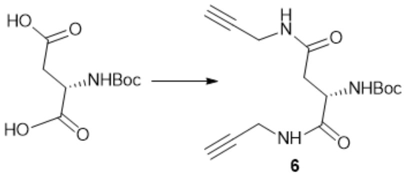

Scheme S2. Reagents and conditions: Boc-L-Aspartic-acid, propargyl amine, DCM, EDC, DMAP, N_2, 0 °C to r.t., o.n.

1,4-bis-(Propargylamido)-Boc-L-Asp (6)

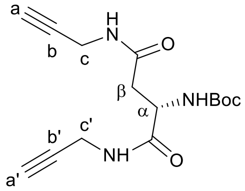

The procedure was adapted from Gao *et al.*[2]; briefly, to a stirred solution of Boc-L-Asp-OH (1 eq, 200 mg, 0.86 mmol) in DCM (10 mL), 1-Ethyl-3-(3-dimethylaminopropyl)carbodiimide (2.2 eq, 362 mg, 1.89 mmol) and 4-dimethylaminopyridine (0.2 eq, 21 mg, 0.17 mmol) were added and stirred at room temperature for 2 hrs. To this mixture, a solution of propargyl amine (2.2 eq, 104 mg, 1.89 mmol) in DCM (2.5 mL) was added at 0 °C. The reaction mixture was allowed to room temperature and stirred under N_2 for 24 hrs. The reaction progress was monitored by TLC (DCM:MeOH 96:4). Upon reaction completion the solvent was evaporated, and the crude product was purified via silica gel chromatography using an elution gradient of 98:2 to 96:4 $CHCl_3$:MeOH to yield **6** (63 %, 165 mg).

R_f 0.3 (DCM:MeOH, 96:4). ^{1}H NMR (CD_3OD, 500 MHz): δ_H 4.32 (1H, m, H_α), 3.86-3.39 (4H, m, H_{c1}, H_{c2}, $H_{c'1}$, $H_{c'2}$), 3.24-3.20 (2H, m, H_a, $H_{a'}$) 2.48 (2H, t, J = 2.56 Hz, $H_{\beta1}$, $H_{\beta2}$), 1.34 (9H, s, -C(CH_3)$_3$); ^{13}C NMR (CD_3OD, 100 MHz,): δ_C 79.9 (C(CH_3)$_3$), 79.0 (C_b, $C_{b'}$), 70.94 (C_a or

$C_{a'}$), 70.81(C_a or $C_{a'}$), 51.49 (C_α), 37.24 (C_β), 28.21 (C_c or $C_{c'}$), 28.13 (C_c or $C_{c'}$), 27.24 (-$C(CH_3)_3$).

(LacAc-t-NH)$_2$-Boc-Asp (7)

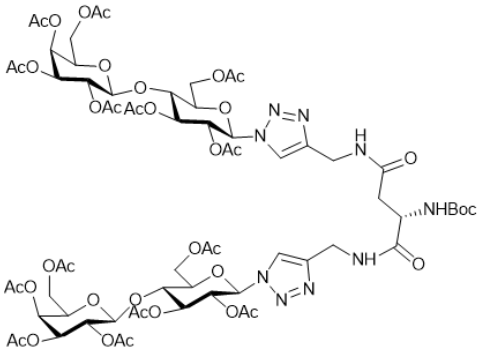

To a solution of peracetylated lactose azide (2 eq, 680 mg, 1.028 mmol,), and Bis(propargylamido)-L-Boc-Asp **6** (1 eq, 158 mg, 0.514 mmol) in MeOH (15 mL), a solution of premixed $CuSO_4$ (0.15 eq, 77 µL 1M, 0.077 mmol,) and Tris buffer (0.30 eq, 154 µL 1M, 0.154 mmol) was added, followed by sodium ascorbate (0.3 eq, 154 µL 1M, 0.154 mmol). The reaction mixture was allowed to stir at room temperature overnight and monitored by TLC (DCM:MeOH, 1:1). Upon reaction completion the solvents were removed under reduced pressure and the mixture was purified by flash chromatography using an elution gradient of 100:0 to 90:10 DCM:MeOH. The product **7** (84 %, 700 mg) was used for the following step without further characterisation.

(Lac-t-NH)$_2$-Boc-Asp (8)

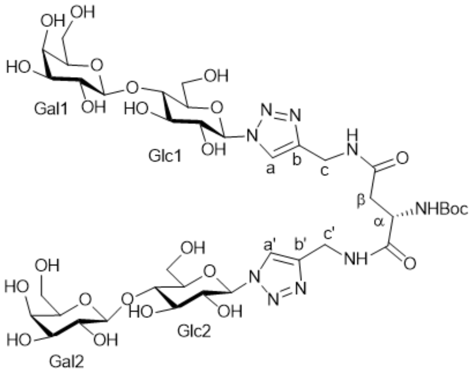

A solution of **7** (1 eq, 700 mg, 0.429 mmol) in dry methanol (10 mL), was treated with sodium methoxide (5 eq, 4.3 mL 0.5 M in methanol) and allowed to react at room temperature overnight until complete conversion. The reaction mixture was neutralised with Dowex H^+ resin, the resin was filtered off and solvent removed under reduced pressure to yield product **8** (98 %, 440 mg). ^{1}H NMR (D_2O, 400 MHz): δ_H 8.04 (1H, s, H_a or $H_{a'}$), 8.01 (1H, s, H_a or $H_{a'}$), 5.67 (1H, d, J = 9.01 Hz, H1-Glc1 or H1-Glc2), 5.66 (1H, d, J = 9.01 Hz, H1-Glc1 or H1-Glc2), 4.42-4.37 (6H, m, H_{c1}, H_{c2}, $H_{c'1}$, $H_{c'2}$, H1-Gal1, H1-Gal2), 3.95 (2H, br t, H2-Glc1, H2-Glc2), 3.88-3.82 (4H, m, H4-Glc1, H4-Glc2, $H_{\beta1}$, $H_{\beta2}$), 3.82-3.74 (9H, m, H3-Glc1, H3-Glc2, H4-Gal1, H4-Gal2, H5-Glc1, H5-Glc2, H5-Gal1, H5-Gal2, H_α), 3.73-3.63 (8H, m, $H6^a$-Gal1, $H6^b$-Gal1, $H6^a$-Gal2, $H6^b$-Gal2, $H6^a$-Glc1, $H6^b$-Glc1, $H6^a$-Glc2, $H6^b$-Glc2), 3.59 (2H, m, H3-Gal1, H3-Gal2), 3.49 (2H, m, H2-Gal1, H2-Gal2), 1.85 (9H, s, OC(CH_3)$_3$); ^{13}C NMR (D_2O, 100 MHz): δ_C 180.57(CO), 144.85 (C_b, $C_{b'}$) 122.99 (C_a or $C_{a'}$), 122.86 (C_a or $C_{a'}$), 102.88 (C1-Gal1, C1-Gal2), 87.24 (C1-Glc1, C1-Glc2), 77.66 (C2-Gal1, C2-Gal 2), 77.28 (C3-Glc1, C3-Glc 2), 75.37 (C5-Glc 1, C5- Glc2), 74.47 (C5 Gal1, C5-Gal2), 72.48 (C4-Gal1, C4-Gal2), 71.96 (C2-Gal1, C2-Gal2), 70.92, (C2-Glc1, C2-Glc2), 61.03 (C6-Glc1, C6-Glc2), 59.68 (C6-Gal1, C6-Gal2), 46.64, 34.39, 27.54 (C_c, $C_{c'}$), 23.17, 8.18(-C(CH$_3$)$_3$). HR ESI-MS found m/z 1040.39 [M-H]$^-$, calcd for $C_{39}H_{63}N_9O_{24}$ 1041.96.

(Lac-t-NH)$_2$-Asp-NH-PEG$_3$-N$_3$ (9)

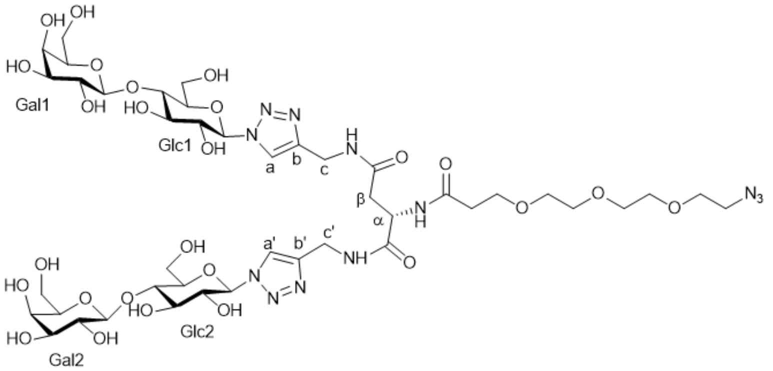

A solution of **8** (20 mg, 0.019 mmol) in dry DCM (3 mL) was treated with TFA (1mL). The reaction mixture was stirred for 20 minutes at room temperature, then the solvents were removed by co-evaporation with DCM (10 mL) (x3) under reduced pressured. The crude product, (Lac-t-NH)$_2$-Asp-NH$_2$ (14 mg, 0.015 mmol) was dissolved in water (0.5 mL) and 1M $NaHCO_3$ was added until neutral pH (ca. 6 drops). A solution of N_3-PEG$_3$-NHS ester in anhydrous DMF (1 mg/mL, 16 µl, 0.05 mmol, 1.1 eq) and diisopropylethylamine (30 µL, 0.17 mmol, 4 eq) were added sequentially. The mixture was allowed to react at room temperature for 3 hrs. The reaction progress was monitored by TLC (iPrOH:NH_4OH:H_2O 6:3:1). MALDI-ToF analysis of the crude reaction mixture showed complete conversion. The solvents were removed under reduced pressure, and the residue was purified by C18 column chromatography to yield **9** (77 %, 14 mg) R_f 0.1. (iPrOH:NH_4OH:H_2O 6:3:1). ^{1}H NMR (D_2O, 400 MHz): δ_H 8.04 (1H, s, H_a or $H_{a'}$), 8.01 (1H, s, H_a or $H_{a'}$), 5.67 (1H, d, J = 9.01 Hz, H1-Glc1 or H1-Glc2), 5.66 (1H, d, J = 9.01 Hz, H1-Glc1 or H1-Glc2), 4.42-4.37 (6H, m, H_{c1}, H_{c2}, $H_{c'1}$, $H_{c'2}$, H1-Gal1, H1-Gal2), 3.95 (2H, br t, H2-Glc1, H2-Glc2), 3.88-3.84 (4H, m, H4-Glc1, H4-Glc2, $H_{\beta 1}$, $H_{\beta 2}$), 3.83-3.75 (8H, m, H3-Glc1, H3-Glc2, H4-Gal1, H4-Gal2, H5-Glc1, H5-Glc2, H5-Gal1, H5-Gal2), 3.75-3.62 (10H, m, H6^a-Gal1, H6^b-Gal1, H6^a-Gal2, H6^b-Gal2, CH_2-PEG, H6^a-Glc1, H6^b-Glc1, H6^a-Glc2, H6^b-Glc2), 3.63-3.56 (8H, m, CH_2-PEG, H3-Gal1 and H3-Gal2), 3.53 (4H, s, CH_2-PEG), 3.49 (2H, m, H2-Gal1, H2-Gal2), 3.39 (2H, m, CH_2-PEG), 2.69 (2H, m, CH_2-PEG), 2.46 (2H, m, CH_2-PEG); ^{13}C NMR (D_2O, 100 MHz): δ_C 180.57(CO), 144.85 (C_b and $C_{b'}$) 122.99 (C_a or $C_{a'}$), 122.86 (C_a or $C_{a'}$), 102.88 (C1-Gal1, C1-Gal2), 87.24 (C1-Glc1, C1-Glc2), 77.66 (C2-Gal1, C2-Gal 2), 77.28 (C3-Glc1, C3-Glc 2), 75.37 (C5-Glc1, C5- Glc2), 74.47 (C5-Gal1, C5-Gal2), 72.48 (C4-Gal1, C4-Gal2), 71.96 (C2-Gal1, C2-Gal2), 70.92, (C2-Glc1, C2-Glc2), 61.03 (C6-Glc1, C6-Glc2), 59.68 (C6-Gal1, C6-Gal2), 46.64, 34.39, 27.54 (C_c, $C_{c'}$). ESI-MS found *m/z* 1169.44 [M-H]$^-$, calcd for $C_{43}H_{70}N_{12}O_{26}$ 1170.45.

(α-2,6'-SALac-t-NH)$_2$-Asp-NH-PEG3-N$_3$ (1)

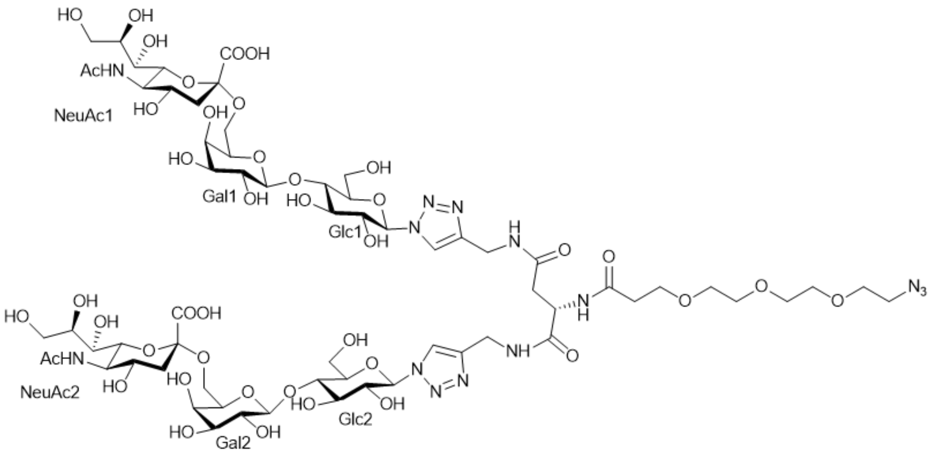

In a 2 mL Eppendorf tube, (Lac-t-NH)$_2$-Asp-NH-PEG$_3$-N$_3$ **9** (20 µL, 50 mM solution in H_2O, 1 mg), CTP (100 µL, 100 mM solution in H_2O), sialic acid (200 µL, 20 mM solution in H_2O), alkaline phosphatase (30 µL, 0.84 mg/ml solution in H_2O), $MgCl_2$ (50 µL, 100 mM solution in H_2O), α2,6-SiaT (19 µL, 10 U/ml solution, 0.19 U), CMP-SA synthetase (2 µL, 50 U/500ml solution, 0.2U), Tris buffer pH 8.3 (250 µL, 200 mM solution), and H_20 (300 µL) were added sequentially. Reaction progress was monitored by TLC (iPrOH:NH_4OH:H_2O 6:3:16:3:1). MALDI-ToF analysis of the crude reaction mixture showed complete conversion after 16 hrs. The enzymatic reaction was performed in seven parallel batches under the same conditions. Upon reaction completion MeOH (500 µl) was added to each tube and the solution was heated at 80 °C for 5 minutes to inactivate the enzyme. The reaction mixtures were allowed to reach room temperature, combined, and transferred into a corning mini microcentrifuge and spun for 10 minutes, at 6000 rpm. The supernatant was recovered and evaporated under reduced pressure. The reaction crude was then purified by NAP-10 column to yield **1** (70 %, 7.3 mg). R_f 0.4, (iPrOH:NH_4OH:H_2O 6:3:16:3:1). ^{1}H NMR (D$_2$O, 400 MHz), 8.06 (1H, s, H_a or $H_{a'}$), 8.04 (1H, s, H_a or $H_{a'}$), 5.69 (1H, d, J = 9.01 Hz, H1-Glc1 or H1-Glc2), 5.67 (1H, d, J = 9.01 Hz, H1-Glc1 or H1-Glc2), 4.42-4.39 (6H, m, H_{c1}, H_{c2}, $H_{c'1}$, $H_{c'2}$, H1-Gal1, H1-Gal2), 3.98 (2H, br t, H2-Glc1, H2-Glc2), 3.92 (2H, m, H4-NeuAc1, H4-NeuAc2), 3.88-3.84 (4H, m, H4-Glc1, H4-Glc2, $H_{\beta 1}$, $H_{\beta 2}$), 3.83-3.74 (20H, m, H3-Glc1, H3-Glc2, H9-NeuAc1, H9-NeuAc2, H4-

Gal1, H4-Gal2, H5-Glc1, H5-Glc2, H5-Gal1, H5-Gal2, H5-NeuAc1, H5-NeuAc2, H6-NeuAc1, H6-NeuAc2, H8-NeuAc1, H8-NeuAc2, H7-NeuAc1, H7-NeuAc2), 3.73-3.62 (24H, m, $H6^a$-Gal1, $H6^b$-Gal1, $H6^a$-Gal2, $H6^b$-Gal2, CH_2-PEG, $H6^a$-Glc1, $H6^b$-Glc1, $H6^a$-Glc2, $H6^b$-Glc2), 3.63-3.54 (6H, m, CH_2-PEG), 3.48 (2H, m, H3-Gal1, H3-Gal2), 3.39 (2H, m, H2-Gal1, H2-Gal2), 2.7 (2H, m, CH_2-PEG), 2.6 (2H, m, CH_2-PEG); 2.65 (2H, dd, J = 13.07 Hz, J = 4.77 Hz, H3eq-NeuAc), 2.47 (2H, m, CH_2-PEG), 1.93 (9H, s, NHCOCH_3), 1.66 (2H, t, J = 12.57 Hz, H3ax-NeuAc). ^{13}C NMR (D_2O, 200 MHz,): δ_C 180.9, 102.88 (C1-Gal1, C1-Gal2), 87.23 (C1-Glc1, C1-Glc2), 77. 15, 68.54, 61.03, 52.72, 50.03. ESI-MS found *m/z* 1753.913 $[M+H]^+$calcd for $C_{65}H_{104}N_{14}O_{42}$·H 1752.64.

Preaparation of α-2,6'-SALac-t-NH-Boc (4)

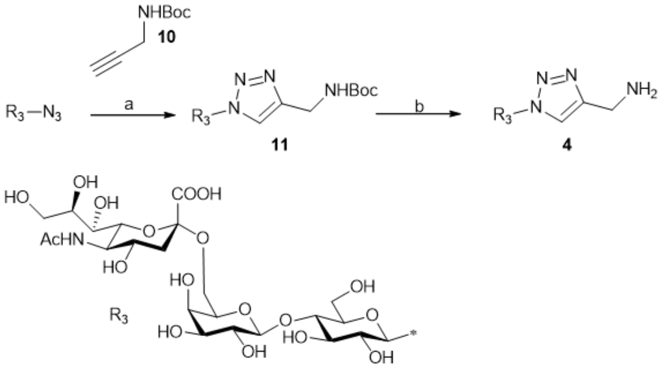

Scheme S3. Preparation of compound **4**. Reagent and conditions: a) Boc-propargyl amine **10**, MeOH, $CuSO_4$, sodium ascorbate, Tris, r.t., o.n.; b) DCM, TFA, r.t., 20 min.

α-2,6'-SALac-t-NH-Boc (11)

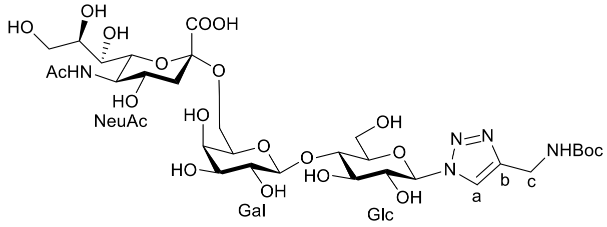

To a solution of α-2,6' sialyl lactose azide (10 mg, 0.015 mmol, 1 eq), and Boc propargyl amine **10** (11 mg, 0.069 mmol, 2 eq), in MeOH (0.3 mL), a solution of premixed $CuSO_4$ (1M, 5 μL, 0.15 eq) and Tris buffer (1M solution, 10 μL, 0.3 eq), was added followed by sodium ascorbate (1M, 10 μL, 0.3 eq). The reaction mixture was allowed to react at room temperature overnight. The reaction was monitored by TLC ($CHCl_3$:MeOH:H_2O 10:6:1). Upon reaction completion the solvents were removed under reduced pressure and the mixture was purified by BioRad High-Q ion exchange cartridge to obtain **11** (97 %, 12 mg). R_f 0.23 ($CHCl_3$:MeOH:H_2O 5:3:0.5). ^{1}H NMR (D_2O, 400 MHz): δ_H 8.05 (1H, s, H_a), 5.68 (1H, d, J = 9.29 Hz, H1-Glc), 4.40 (1H, d, J = 7.80 Hz, H1-Gal), 4.29 (2H, s, H_{c1}, H_{c2}), 3.98 (1H, m, H2-Glc), 3.92 (1H, dd, J = 8.44, J = 9.79 Hz, H4-NeuAc), 3.88-3.51 (14H, m, H3-Glc, H9-NeuAc, H4-Glc, H5-Glc, H4-Gal, H5-Gal, H5-NeuAc, H6-NeuAc, H7-NeuAc, H8-NeuAc, H6^a-Gal, H6^b-Gal, H6^a-Glc, H6^b-Glc), 3.51-3.44 (2H, m, H2-Gal, H3-Gal), 2.64 (1H, dd, J = 12.36 Hz, J = 4.62 Hz, H3eq-NeuAc), 1.94 (3H, s, NHCOCH_3), 1.66 (2H, t, J = 12.24 Hz, H3ax-NeuAc), 1.33 (9H, s, OC(CH_3)$_3$). ^{13}C NMR (D_2O, 100 MHz): δ_c 174.84 (CO), 173.44 (C1-NeuAc), 158.2 (C_b), 122.8 (C_a), 103.4 (C1-Gal), 100.25 (C2-NeuAc), 87.09 (C1-Glc), 78.57 (C4-Glc), 77.50 (C3-Glc), 74.72 (C5-Glc), 73.73 (C6-NeuAc), 72.49 (C8-NeuAc), 72.34 (C5-Gal), 72.33 (C4-Gal), 72.2 (C2-Glc), 70.75 (C4-NeuAc), 70.64 (C2-Gal), 68.51 (C7-NeuAc), 68.42 (C3-Gal), 68.37 (C4-Gal), 63.62 (C9-NeuAc), 63.04 (C4-NeuAc), 62.62 (C6-Glc), 59.85 (C6-Gal), 51.75 (C5-NeuAc), 35.34 (C_c) 40.08 (C3-NeuAc), 22.02 (NHCOCH_3), 27.57 (OC(CH_3)$_3$). ESI-MS found *m/z* 852.27 [M+K]$^+$, calcd. for $C_{31}H_{51}N_5O_{20}$·K 852.86.

α-2,6'-SALac-t-NH$_2$ (4)

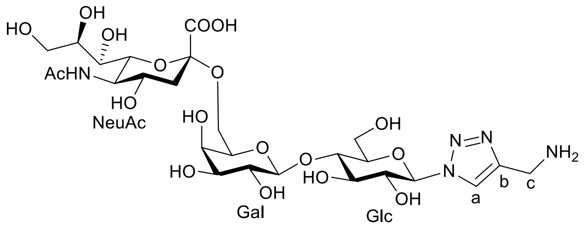

A solution of **11** (13 mg, 0.014 mmol) in dry DCM (1.5 mL) was treated with TFA (0.5 mL). The reaction mixture was allowed to react for 20 minutes at room temperature. The reaction was monitored by TLC ($CHCl_3$:MeOH:H_2O 10:6:1) and when complete the solvents were concentrated under reduced pressure and co-evaporated with DCM (10 mL) (x3). The product **4** was obtained pure (95 %, 13 mg). R_f 0.14 ($CHCl_3$:MeOH:H_2O 10:6:1). ^1H NMR (D_2O, 400 MHz: δ_H 8.20 (1H, s, Ha), 5.68 (1H, d, J = 9.29 Hz, H1-Glc), 4.34 (1H, d, J = 7.63 Hz, H1-Gal), 4.23 (2H, s, H_{c1}, H_{c2}), 3.94 (2H, br H2-Glc, H4-NeuAc), 3.85-3.70 (14H, m, H3-Glc, H9-NeuAc, H4-Glc, H5-Glc, H4-Gal, H5-Gal, H5-NeuAc, H6-NeuAc, H8-NeuAc, H7-NeuAc, $H6^a$-Gal, $H6^b$-Gal, $H6^a$-Glc, $H6^b$-Glc), 3.51-3.47 (2H, m, H2-Gal, H3-Gal), 2.56 (1H, dd, J = 12.79 Hz, J = 4.75 Hz, H3eq-NeuAc), 1.96 (3H, s, NHCO*CH₃*), 1.66 (2H, t, J = 12.40 Hz, H3ax-NeuAc). ^{13}C NMR (D_2O, 100 MHz): δc 174.85 (NH*C*OCH₃), 170.44 (C1-NeuAc), 124.6 (C_a), 103.15 (C1-Gal), 98.70 (C2-NeuAc), 87.08 (C1-Glc), 78.65 (C4-Glc), 77.45 (C3-Glc), 74.06 (C5-Glc), 73.52 (C6-NeuAc), 74.60 (C8-NeuAc), 72.34 (C5-Gal), 72.33 (C4-Gal), 71.86 (C2-Glc), 70.75 (C4-NeuAc), 70.64 (C2-Gal), 68.51 (C7-NeuAc), 68.42 (C3-Gal), 68.37 (C4-Gal), 63.62 (C9-NeuAc), 63.04 (C4-NeuAc), 67.39 (C6-Gal), 62.99 (C6-Glc), 59.73 (C5-NeuAc), 51.59 (C_c), 39.01 (C3-NeuAc), 22.02 (NHCO*C*H₃). ESI-MS found *m/z* 714.29 $[M+H]^+$, calcd for $C_{23}H_{43}N_5O_{18}\cdot H$ 714.65.

Preparation of α-2,3'-SALac-t-NH-Boc (5)

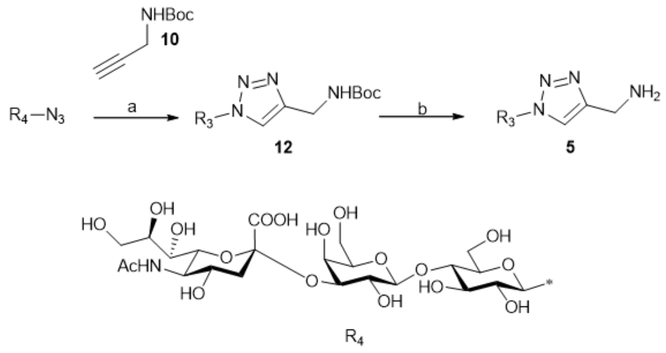

Scheme S4. Preparation of compound **5**. Reagent and conditions: a) Boc-propargyl amine **10**, MeOH, $CuSO_4$, sodium ascorbate, Tris, r.t., o.n.; b) DCM, TFA, r.t., 20 min.

α-2,3'-SALac-t-NH-Boc (12)

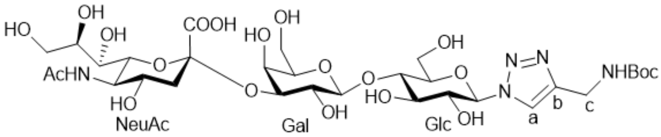

The procedure was adapted from reported literature. To a solution of α2,3' sialyllactose azide [3,4] (8 mg, 0.012 mmol, 1 eq), and Boc propargyl amine **10** (4 mg, 0.024 mmol, 2 eq), in MeOH (1/1) (0.4 mL), a solution of premixed $CuSO_4$ (1M, 5 µL, 0.15 eq) and Tris buffer (1M solution, 14 µL, 0.3 eq), was added followed by sodium ascorbate (1M, 3 µL, 0.3 eq). The reaction mixture was allowed to react at room temperature overnight. The reaction was monitored by TLC ($CHCl_3$:MeOH:H_2O 10:6:1) and when complete the solvents were removed under reduced pressure and the mixture was purified by BioRad High-Q ion exchange cartridge. The product **12** was obtained pure (99 %, 10 mg). R_f 0.38 ($CHCl_3$:MeOH:H_2O 5:3:0.5). ^{1}H NMR (D_2O, 400 MHz): δ_H 8.05 (1H, s, H_a), 5.68 (1H, d, *J* =9.29 Hz, H1-Glc), 4.51 (1H, d, *J* = 7.80 Hz, H1-Gal), 4.29 (2H, s, H_{c1}, H_{c2}), 4.05 (1H, dd, *J*= 9.94 Hz, *J*= 3,14 Hz, H2-Glc) , 3.96 (1H, t, *J* = 9.62, H4-NeuAc), 3.93-3.37 (16H, m, H3-Glc, H9-NeuAc, H4-Glc, H5-Glc, H4-Gal, H5-Gal, H5-NeuAc, H6-NeuAc, H7-NeuAc, H8-NeuAc, $H6^a$-Glc, $H6^b$-Glc, $H6^a$-Gal, $H6^b$-Gal, H2-Gal, H3-Gal), 2.64 (1H, dd, *J* = 12.36 Hz, *J* = 4.62 Hz, H3eq-NeuAc), 1.94 (3H, s, NHCOCH_3), 1.66 (2H, t, *J* = 12.24 Hz, H3ax-NeuAc), 1.33 (9H, s, OC(CH_3)$_3$). ^{13}C NMR (D_2O, 100 MHz: δ_c 174.84 (NH*C*OCH$_3$), 173.44 (C1-NeuAc), 157.3 (C_b), 120.9 (C_a), 102.6 (C1-Gal), 93.20 (C2-NeuAc), 87.35 (C1-Glc), 77.6 (C4-Glc), 75.6 (C2-Glc), 75.16 (C3 Glc), 75.27 (C5-Glc), 74.39 (C6-NeuAc), 72.87 (C8-NeuAc), 72.79(C5-Gal), 71.79 (C4-Gal), 69.42 (C4-NeuAc), 68.0 (C2-Gal), 68.5 (C7-NeuAc), 67.2 (C3-Gal), 67.5 (C4-Gal), , 62.58 (C6-Glc), 62.06 (C6-Gal), 59.7 (C9-NeuAc), 51.69 (C4-NeuAc), 35.47 (C_c), 39.93 (C3-NeuAc), 22.02

(NHCOCH_3), 27.57 (OC(CH_3)$_3$). ESI-MS found *m/z* 814.32 $[M+H]^+$, calcd for $C_{23}H_{43}N_5O_{18}$·H 814.72.

α-2,3'-SALac-t-NH₂ (5)

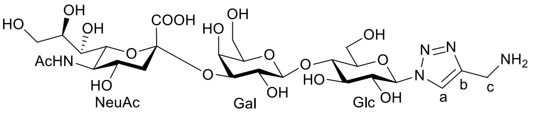

A solution of **12** (5.70 mg, 0.061 mmol) in dry DCM (1 mL) was treated with TFA (250 uL). The reaction mixture was stirred for 20 minutes at room temperature, then the solvents were removed by co-evaporation with DCM (10 mL) (x3) under reduced pressured. The product **5** was obtained clean and used without further purification (96 %, 4.2 mg). R*f* 0.23 ($CHCl_3$:MeOH:H_2O 5:3:0.5). ^{1}H NMR (D_2O, 400 MHz): δ_H 8.05 (1H, s, H_a), 5.68 (1H, d, *J* = 9.16 Hz, H1-Glc), 4.51 (1H, d, *J* = 7.73 Hz, H1-Gal), 4.29 (2H, s, H_{c1}, H_{c2}), 4.06 -3.95 (2H br, m, H2-Glc, H4-NeuAc), 3.94-3.55 (16H, m, H3-Glc, H9-NeuAc, H4-Glc, H5-Glc, H4-Gal, H5-Gal, H5-NeuAc, H6-NeuAc, H8-NeuAc, H7-NeuAc, H6^a-Glc, H6^b-Glc, H6^a-Gal, H6^b-Gal), 3.81 (2H, m, H2-Gal, H3-Gal), 2.69 (1H, dd, *J* = 12.62 Hz, *J* = 4.17 Hz, H3eq-NeuAc), 1.95 (3H, s, NHCOCH_3), 1.73 (2H, t, *J* = 12.26 Hz, H3ax-NeuAc). ^{13}C NMR (D_2O, 100 MHz,): δ_c 174.84 (NH*C*OCH$_3$), 173.44 (C1-NeuAc), 157.3 (C_b), 120.9 (C_a), 102.6 (C1-Gal), 93.20 (C2-NeuAc), 87.35 (C1-Glc), 77.6 (C4-Glc), 75.6 (C2-Glc), 75.16 (C3 Glc), 75.27 (C5-Glc), 74.39 (C6-NeuAc), 72.87 (C8-NeuAc), 72.79(C5-Gal), 71.79 (C4-Gal), 69.42 (C4-NeuAc), 68.0 (C2-Gal), 68.5 (C7-NeuAc), 67.2 (C3-Gal), 67.5 (C4-Gal), 62.58 (C6-Glc), 62.06 (C6-Gal), 59.7 (C9-NeuAc), 51.69 (C4-NeuAc), 35.47 (C_c) 39.93 (C3-NeuAc), 22.02 (NHCOCH_3). ESI-MS found *m/z* 714.29 $[M+H]^+$, calcd for $C_{23}H_{43}N_5O_{18}$·H 714.65.

Preparation of (α-2,6'-SALac-t-NH-propanamido)₂-NH-PEG3-N₃ (2)

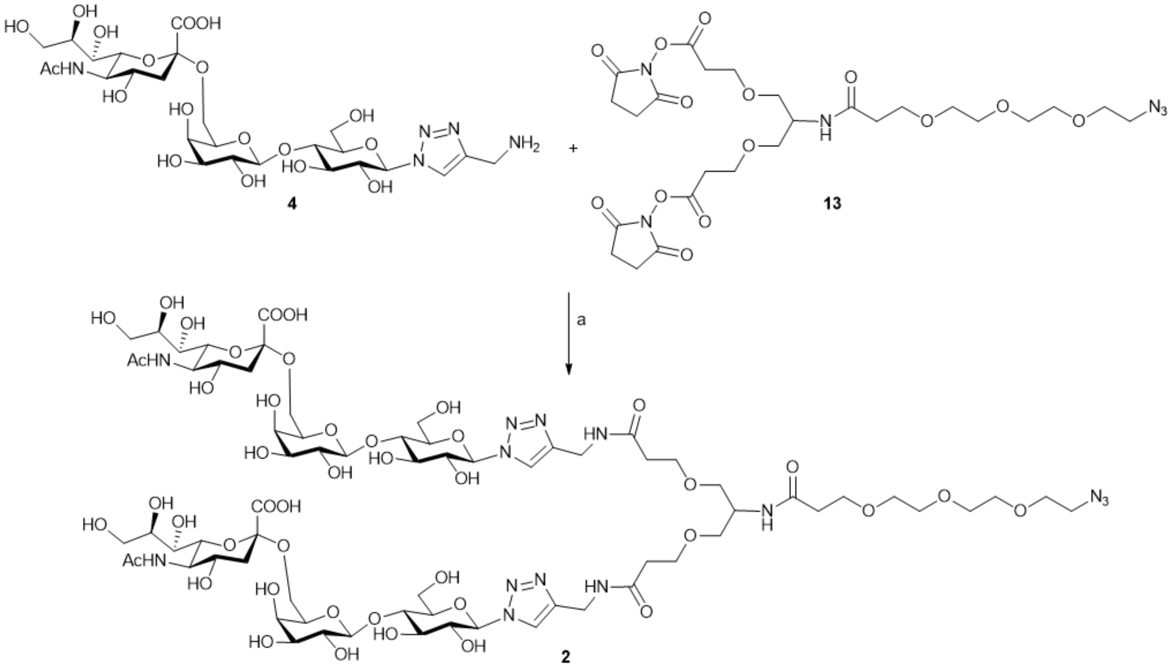

Scheme S5. Bis-NHS-PEG3 linker **13**, **4,** DMF, 100 mM HEPES pH 8.0, 0°C, 24 hrs.

(α-2,6’-SALac-t-NH-propanamido)$_2$-NH-PEG3-N_3 (2)

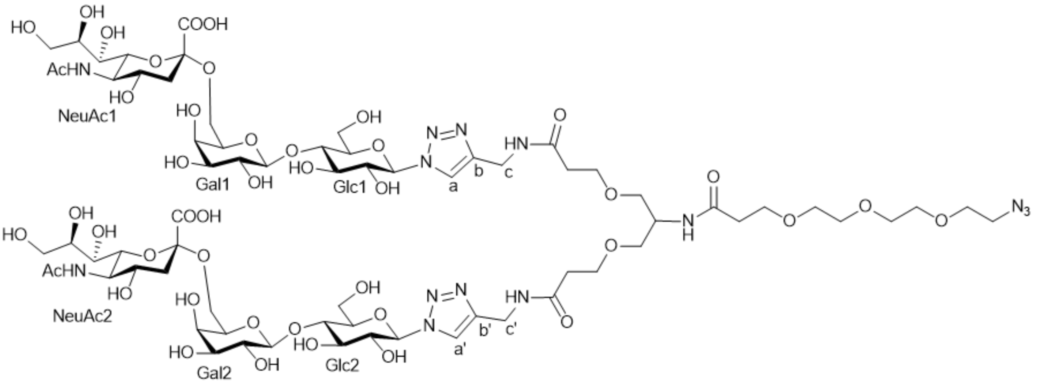

2,6’SALac-t-CH_2-NH_2 **4** (8.5 mg, 0.011 mmol, 3 eq) was dissolved in 50 mM HEPES buffer pH 8.0 (200 μL). The solution was cooled down to 0 °C and 2-(N_3-PEG$_3$-amido)-1,3-bis(NHS ester) **13** (1 eq, 2.6 mg, 0.003mmol, 10 μL) was added. The reaction was allowed to reach room temperature, and stirred for further 36 hrs. The reaction progress was monitored by TLC (iPrOH:NH_4OH:H_2O 6:3:1). MALDI-ToF analysis of the crude reaction mixture showed the presence of the mono- and di-functionalised α-2-6’-sialic acid glycans. The solvent was removed under reduce pressure, and the residue was purified by C18 column and SPE ENVI

CARB column to yield the target **2** (12 %, 6.8 mg). R*f* 0.4 (iPrOH:NH_4OH:H_2O 6:3:1). ^1H NMR (D_2O, 400 MHz): δ_H 8.08 (2H, s, $H_{a,}$ $H_{a'}$), 5.69 (2H, d, *J* = 9.30 Hz, H1-Glc1, H1-Glc2), 4.47-4.37 (6H, m, H_{c1}, H_{c2}, $H_{c'1}$, $H_{c'2}$, H1-Gal1, H1-Gal2), 4.05 (1H, m, H_g), 3.98 (2H, br t, H2-Glc1, H2-Glc2), 3.92 (2H, m, H4-NeuAc1, H4-NeuAc1), 3.94-3.72 (22H, m, H4-Glc1, H4-Glc2, ,H3-Glc1, H3-Glc2, H9-NeuAc1, H9-NeuAc2, H4-Gal, H4-Gal2, H5-Glc1, H5-Glc2, H5-Gal1, H5-Gal2, H5-NeuAc1, H5- NeuAc2, H6-NeuAc1, H6-NeuAc2, H8-NeuAc1, H8-NeuAc2, H7-NeuAc1, H7-NeuAc2), 3.72-3.52 (20H, m, H6^a-Gal1, H6^b-Gal1 H6^a-Gal2, H6^b-Gal2, H_e, $H_{e'}$, H_d and $H_{d'}$, CH_2-PEG, H6^a-Glc1, H6^b-Glc1, H6^a-Glc2, H6^b-Glc2) 3.52-3.45 (4H m, H3-Gal1, H3-Gal2, H2-Gal1, H2-Gal2), 3.45-3.36 (6H, m, CH_2PEG, H_f and $H_{f'}$), 2.64 (2H, dd, *J* = 10.62 Hz, *J* = 4.74 Hz, H3eq-NeuAc1, H3eq-NeuAc2), 2.46 (6H, m, CH_2 PEG), 1.93 (9H, s), 1.66 (2H, t, *J* = 12.25 Hz, H3ax-NeuAc1, H3ax-NeuAc2). HR-ESI-MS found *m/z* 1856.71 $[M+H]^+$, calcd for $C_{70}H_{116}N_{12}O_{44}$·H 1856.71.

Preparation of (α-2,3'-SALac-t-NH-propanamido)$_2$-NH-PEG3-N_3 (3) and (mono-α-2,6'-SALac-t-NH-propanamido)-NH-PEG3-N_3 (17)

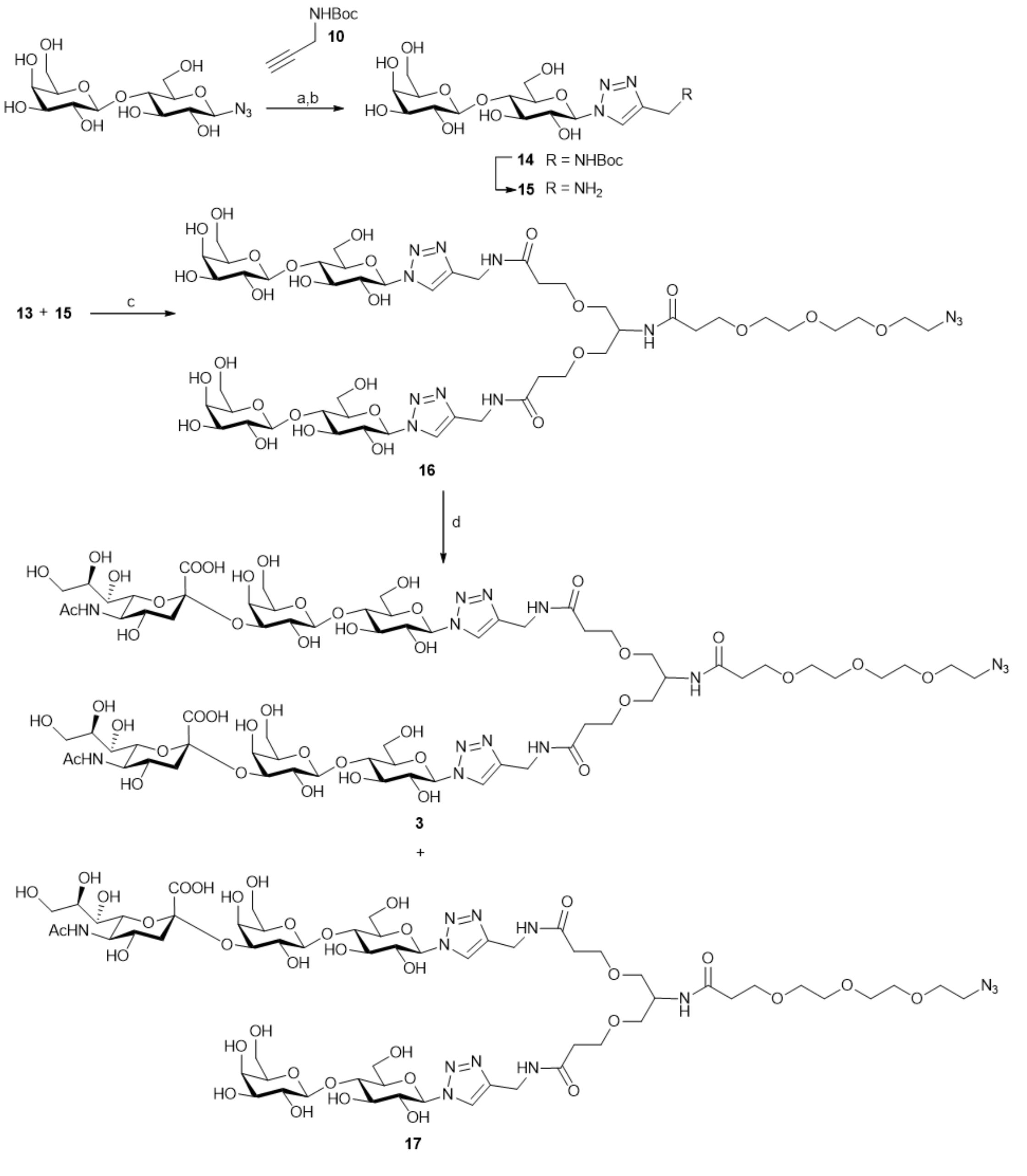

Scheme S6. Synthetic scheme for the preparation of **3** and **17.** Reaction conditions: a) **10**, THPTA, $CuSO_4$, sodium ascorbate, DMF:H_2O (1:1), b) (DCM:TFA 1:1); c) Di-isopropylethylamine, DMF, rt, 24 h; d) Fetuin, TcTS (0.2 mg/mL), Tris-HCl (pH = 7.8).

β-Lac-(4-Boc-aminomethyl-[1,2,3]-triazole) (14)

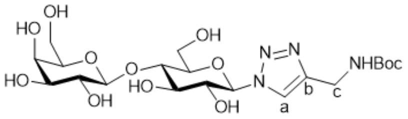

Lactosyl azide (15 mg, 41 µmol) and N-Boc propargyl amine **10** (22 mg, 143 µmol) were dissolved in H_2O: DMF 1:1 (0.4 mL). Tris-hydroxypropyltriazolylmethyl amine (THPTA) (14 mg, 32 µmol) was premixed with $CuSO_4$ (3 mg, 12 µmol) and were added to the reaction mixture. Sodium ascorbate (1 mg, 5 µmol) was then added and the reaction mixture was stirred at room temperature overnight. Solvent was removed under reduced pressure. The residue was dissolved in H_2O and was stirred with IWT TMD-8 ion exchange resin for 15 minutes. The mixture was filtered, concentrated under reduced pressure, and the residue was purified by gel permeation chromatography (30 mg injection, TSK gel Toyopearl HW-40S, 2.2 × 90 cm) in water at 0.5 mL min^{-1}, to yield **14** (47 %, 10 mg); R_f 0.53 (6:3:1 IPA/NH_4OH/H_2O); (400 MHz, D_2O): δ_H 8.13 (1H, s, H_a), 5.77 (1H, d, J = 9.2 Hz, H1-Glc), 4.52 (1H, d, J = 7.7 Hz, H1-Gal), 4.38 (2H, s, H_{c1}, H_{c2}), 4.06 (1H, t, J = 8.8 Hz, H2-Glc), 4.00-3.74 (9H, m, H3-Glc, H4-Glc, H5-Glc, $H6^a$-Glc, $H6^b$-Glc, H4-Gal, H5-Gal, $H6^a$-Gal, $H6^b$-Gal), 3.69 (1H, dd, J = 10.1 Hz, J = 3.4 Hz, H3-Gal), 3.59 (1H, dd, J = 7.7 Hz, J = 10.1 Hz, H2-Gal), 1.42 (9H, s, OC(CH_3)$_3$). ^{13}C NMR (100 MHz, D_2O): δ_C 158.1 (C=O), 122.6 (C_a), 103.2 (C1-Gal), 87.5 (C1-Glc), 77.68 (C4-Glc), 77.29 (C3-Glc), 75.38 (C5-Glc), 74.49 (C3-Gal), 72.49 (C5-Gal), 72.0 (C2-Glc), 70.9 (C2-Gal), 68.5 (C4-Gal), 60.9 (C6-Glc), 59.8 (C6-Gal), 35.2 (C_c), 27.3 (C(CH_3)$_3$). ESI-MS found *m/z* 545.3 $[M+Na]^+$, calcd for $C_{20}H_{34}N_4O_{12}$·Na 545.22.

β-Lac-(4-aminomethyl-[1,2,3]-triazole) (15)

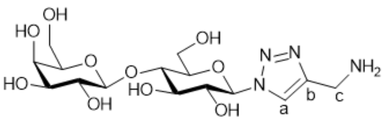

A solution of **14** (10 mg, 0.019 mmol) in dry DCM (3 mL) was treated with TFA (0.4 mL) and the mixture was stirred for 20 minutes at room temperature. The reaction was monitored by TLC ($CHCl_3$:MeOH:H_2O 10:6:1), once complete, the solvents were concentrated under

reduced pressure and co-evaporated with DCM (10 mL) (x3). The product **15** was used for the next step without further purification. (400 MHz, D_2O): δ_H 8.21 (1H, s, H_a), 5.68 (1H, d, J = 9.2 Hz, H1-Glc), 4.36 (1H, d, J = 7.7 Hz, H1-Gal), 4.23 (2H, s, H_c), 3.93 (1H, t, J = 8.8 Hz, H2-Glc), 4.00-3.74 (9H, m, H3-Glc, H4-Glc, H5-Glc, $H6^a$-Glc, $H6^b$-Glc, H4-Gal, H5-Gal, $H6^a$-Gal, $H6^b$-Gal), 3.53 (1H, dd, J = 10.1 Hz, J= 3.3 Hz, H3-Gal), 3.43 (1H, dd, J= 7.7 Hz, J =10.1 Hz, H2-Gal). ^{13}C NMR (100 MHz, D_2O): δ_C 146.1 (C_b), 125 (C_a), 102.8 (C1-Gal), 87.3 (C1-Glc), 77.7 (C4-Glc), 75.5 (C5-Gal), 74.5 (C3-Glc), 72.5 (C3-Gal), 72.0 (C2-Glc), 70.9 (C2-Gal), 68.5 (C4-Gal), 61.1 (C6-Glc), 59.8 (C6-Gal), 34.0 (C_c). ESI-MS found *m/z* 421.15 $[M-H]^-$, calcd for $C_{15}H_{26}N_4O_{10}$ 422.39.

(Lac-t-NH-propanamido)$_2$-NH-PEG3-N_3 (16)

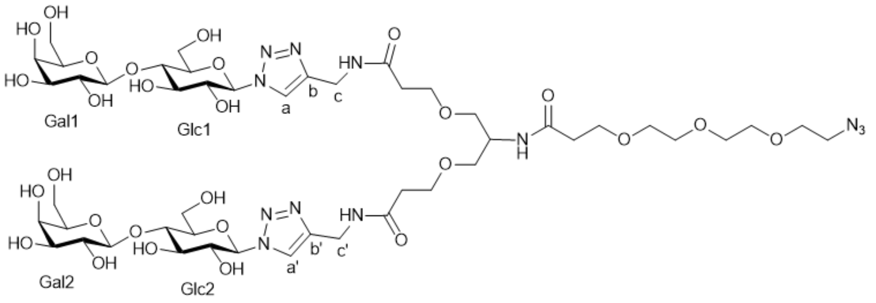

A solution of Azido-PEG$_3$-NHS ester **13** in anhydrous DMF (5 mg, 16 µL, 0.0078 mmol, 1.0 eq.) and Diisopropylethylamine (30 µL, 0.17 mmol, 4 eq.) were added sequentially to a solution of **15** (8 mg, 0.019 mmol, 2.4 eq.) in DMF (0.4 mL). The reaction mixture was left to react at room temperature for 24 h. The reaction progress was monitored by TLC (iPrOH:NH_4OH:H_2O 6:3:1). MALDI-ToF analysis of the crude reaction mixture showed complete conversion. The solvents were removed under reduced pressure, and the residue was purified by gel permeation chromatography to yield **16** (45 %, 9.7 mg). (iPrOH:NH_4OH:H_2O 6:3:1). δ_H ^1H NMR (400 MHz, D_2O): 8.15 (2H, s, H_a and $H_{a'}$), 5.76 (2H, d, J = 9.1 Hz, H1-Glc1 and H1-Glc2), 4.52-4.49 (6H, m, H1-Gal1, H1-Gal2, H_c and $H_{c'}$), 4.12 (2H, t, J = 5.6 Hz, H_a, $H_{a'}$), 4.04 (2H, m, H2-Gal1, H2-Glc2), 3.95-3.64 (34H, m), 3.58 (2H, dd, J = 9.9, J= 7.8 Hz, H2-Gal1, H2-Gal2),

3.48 (6H, dd, J = 11.3 Hz, J = 5.2 Hz), 3.43-3.38 (6H, m, H_{c1}, H_{c2}, $H_{c'1}$, $H_{c'2}$, H1-Gal1, H1-Gal2), 2.51 (6H, dt, J = 12.2 Hz, J = 6.1 Hz). ^{13}C NMR (100 MHz, D_2O): δ_C 180.9 (C=O), 173 (C=O), 144 (C=O), 145.0 (C_b, $C_{b'}$), 123.1 (C_a, $C_{a'}$), 102.88 (C1-Gal1, C1-Gal2), 87.3 (C1-Glc1, C1-Glc2), 77.8 (C4-Glc1, C4-Glc2), 77.1 (C3-Glc1, C3-Glc2), 75.5 (C5-Glc1, C5-Glc2), 74.5 (C3-Glc1, C3-Glc2), 72.5 (C3-Gal1, C3-Gal2), 72.0 (C2-Glc1 or C2-Glc2), 71.8 (C2-Glc2 or C2-Glc1), 70.9 (C2-Gal1, C2-Gal2), 68.5, 61.1 (C6-Glc1, C6-Glc2), 59.8 (C6-Gal1, C6-Gal2), 52.7, 50.2, 34.0 (C_c, $C_{c'}$). ES-MS: found m/z $[M+Na]^+$ 1295.82 calcd for $C_{48}H_{80}N_{12}O_{28}\cdot Na$ 1296.20.

(α-2,3'-SALac-t-NH-propanamido)$_2$-NH-PEG3-N$_3$ (3)

and (mono-α-2,3'-SALac-t-NH-propanamido)-NH-PEG3-N$_3$ (17)

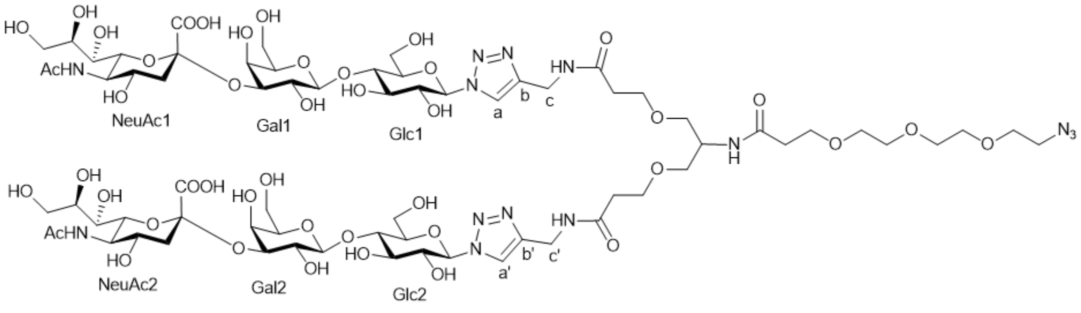

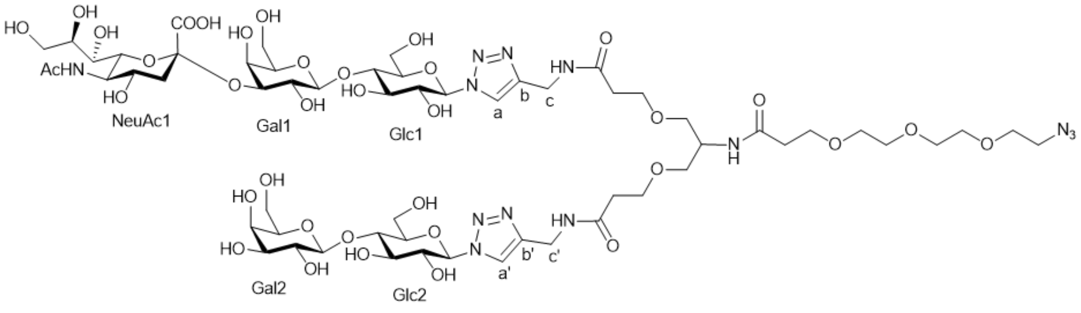

The enzymatic transformation was performed as previously reported.[5,6] In a 2 mL Eppendorf tube, 2-(N$_3$-PEG3-amido)-1,3-bis Lac **7** (3 mg, 2.35 mmol, 1.0 eq.) and Fetuin (70 mg, 23 eq.) were dissolved in 50 mM Tris-HCl buffer pH 7.8 (0.60 mL). *Trypanosoma cruzi trans*-sialidase (TcTS) (0.2 mg/mL) was added to the mixture and the reaction was incubated at 30 °C for 8

hours. The reaction was monitored by TLC analysis (iPrOH:NH_4OH:H_2O 6:3:1). MALDI-ToF analysis of the crude reaction mixture showed the presence of the di- and mono-functionalised α-2,3' sialylated glycan **3** and **17**. The enzymatic reaction was performed in seven parallel batches under the same conditions. Upon reaction completion, EtOH (0.5 mL) was added to each tube and the solution was cooled at 0 °C for 10 minutes to inactivate and precipitate the enzyme. The reaction mixtures were combined and transferred into a corning mini microcentrifuge and spun for 10 minutes, at 6000 rpm. The supernatant was recovered and evaporated under reduced pressure. The reaction crude was then purified by DEAE-weak anion exchange column from Bio-Rad using ammonium bicarbonate (0-200 mM) to yield **3** (40 %, 3.5 mg) and **17 (**20 %, 2.3 mg)

Compound **3**. R_f 0.4 (iPrOH:NH_4OH:H_2O 6:3:1). ^1H NMR (800 MHz, D_2O): δ_H 8.10 (2H, s, H_a, $H_{a'}$), 5.67 (2H, d, J = 9.2 Hz, H1-Glc1, H1-Glc2), 4.50 (2H, d, J = 7.8 Hz, H1-Gal1, H1-Gal2), 4.42 (4H, s, H_c, $H_{c'}$), 4.04 (2H, dd, J = 9.8 Hz, J = 3.1 Hz, H_a, $H_{a'}$), 3.96-3.93 (2H, m, H2-Glc1, H2-Gal2), 3.89-3.86 (2H, m, NeuAc1-H9, NeuAc2-H9, NeuAc1-H4, NeuAc2-H4), 3.82- 3.74 (20H, m), 3.65-3.53 (32H, m, NeuAc1-H5, NeuAc2-H5), 3.48 (4H, m), 3.39- 3.28 (2H, m), 2.80 (1H, dd), 2.67 (2H, dd, J = 12.5 Hz, J = 4.7 Hz, NeuAc1-$H3_{eq}$, NeuAc2-$H3_{eq}$), 2.43 (2H, t, J = 6.0 Hz), 2.41 (1H, t, J = 6.2 Hz, NeuAc1-$H3_{ax}$ or NeuAc2-$H3_{ax}$), 1.94 (6H, s, 2 × NHAc), 1.71 (1H, t, J = 12.1 Hz, NeuAc1-$H3_{ax}$ or NeuAc2-$H3_{ax}$); ^{13}C NMR (200 MHz, D_2O): δ_C 180.9 (C=O), 177.0 (C=O), 173.9 (C=O), 173.8 (C=O), 145.0 (C_b, $C_{b'}$), 144.1 (C=O), 144.5 (C=O), 122.8 (C_a, $C_{a'}$), 102.8 (C1-Gal1), 102.6 (C1-Gal2), 99.6, 88.2, 87.1 (C1-Glc1, C1-Glc2), 77.9 (C4-Glc1, C4-Glc2), 75.3 (C5-Glc1, C5-Glc2), 73.4, 72.3 (C3-Gal1, C3-Gal2), 78.5, 70.2 (C2-Gal1, C2-Gal2), 69.1, 68.6, 67.9, 66.7, 61.1 (C6-Glc1, C6-Glc2), 59.8 (C6-Gal1, C6-Gal2), 51.8, 50.1, 39.6 (C3-NeuAc1, C3-NeuAc2), 34.0 (C_c, $C_{c'}$), 22.1 (2 × *C*H_3, NHAc). ESI-MS found (ESI^-) *m/z* 1856.71 $[M-H]^-$ calcd 1855.72 $C_{70}H_{114}N_{14}O_{44}$

Compound **17.** R_f 0.35 (iPrOH:NH_4OH:H_2O 6:3:1). ^1H NMR (500 MHz, D_2O): δ_H 8.06 (2H, s, H_a and $H_{a'}$), 5.68 (2H, d, *J* = 9.2 Hz, H1-Glc1, H1-Glc2), 4.49 (2H, d, *J* = 7.8 Hz, 1H-Gal1, H1-Gal2) , 4.45 (2H, s, H_c), 4.42 (2H, s, $H_{c'}$), 4.11 (1H, t, *J* = 5.8 Hz, H_a, $H_{a'}$), 4.04 (2H, dd, *J* = 9.8 Hz), 3.95 (2H, t, *J* = 9.1 Hz, H2-Glc1, H2-Gal2), 3.89-3.83 (4H, m, NeuAc-H9, NeuAc-H4, NeuAc-H5), 3.83 -3.73 (12H, m), 3.68 (29H, m, NeuAc-H6), 3.64 – 3.56 (13H, m), 3.54 – 3.43 (8H, m, NeuAc-H7, NeuAc-H8), 3.40 (5H, q, *J* = 5.1 Hz), 2.67 (1H, dd, *J* = 12.4 Hz, *J* = 4.6 Hz, NeuAc-$H3_{eq}$) 2.44 (11H, m), 1.94 (3H, s, CH_3, NHAc), 1.71 (1H, t, *J* = 12.1 Hz, NeuAc-$H3_{ax}$). ^{13}C NMR (200 MHz, D_2O): δ_C 180.9 (C=O), 177.0 (C=O), 173.9 (C=O), 173.8 (C=O), 145.0 (C_b, $C_{b'}$), 144.1 (C=O), 122.8 (C_a, $C_{a'}$), 102.8 (C1-Gal1), 102.6 (C1-Gal2), 99.6, 88.2, 87.1 (C1-Glc1, C1-Glc2), 77.9 (C4-Glc1, C4-Glc2), 75.3 (C5-Glc1, C5-Glc2), 73.4, 72.3 (C3-Gal1, C3-Gal2), 70.2 (C2-Gal1, C2-Gal2), 69.1, 68.6, 67.9, 66.7, 61.1 (C6-Glc1, C6-Glc2), 59.8 (C6-Gal1, C6-Gal2), 51.8, 50.1, 39.6 (NeuAc-C3), 34.0 (C_c, $C_{c'}$), 22.1 (*C*H_3, NHAc). ESI-MS found (ESI^-) *m/z* 1564.62 $[M-H]^-$ calcd for $C_{59}H_{97}N_{13}O_{36}$ 1564.47.

Polymerisation of *N*-(2-hydroxyethyl)acrylamide (HEA) using 2-(dodecylthiocarbonothioylthio)-2-methylpropanoic acid pentafluorophenyl ester (PFP-DMP) chain transfer agent (CTA).

A vial was charged with 2-(dodecylthiocarbonothioylthio)-2-methylpropanoic acid pentafluorophenyl ester (PFP-DMP) (368.7 mg, 0.69 mmol, 1 eq), *N*-(2-hydroxyethyl) acrylamide (HEA) (2 g, 17.4 mmol, 25 eq), ACVA (39 mg, 0.14 mmol, 0.2 eq), and 8 mL of dioxane/methanol mixture (1:1) with 150 µL of mesitylene as an internal NMR standard. An aliquot was withdrawn for determination of monomer conversion by ^1H NMR in $CDCl_3$. The vial was then sealed and deoxygenated using N_2 for 15 mins. Polymerised at 70 °C for 90 mins. The polymerisation was quenched by exposing the vial to air and submerging it into liquid N_2. An aliquot was withdrawn for determination of monomer conversion by ^1H NMR spectroscopy

in MeOD. The polymer was precipitated into diethyl ether from methanol twice to yield a yellow polymer product that was further dried under vacuum. Conversions were calculated using ^{1}H NMR spectroscopy by comparing the integrations of the HEA monomer signals (δ 5.67 ppm) with those of the corresponding signals of mesitylene (δ 6.78 ppm). Mn,NMR was calculated by end-group analysis by comparing the integrations of the –CH3 signals (δ 0.92 ppm) of dodecyl end-group with those of the corresponding signals of the polymer (δ 2.22–2.04 ppm). H NMR (400 MHz, CD_3OD): δ (ppm) 8.15–8.03 (br m, N***H*** of PHEA side chain), 3.89–3.13 (br m, NH–C***H2*** and C***H2***–OH of PHEA side chain), 2.35– 2.05 (br m, C***H*** of PHEA backbone), 1.85–1.31 (br m, C***H2*** of PHEA backbone), 0.92 (t, 3H, CH2–C***H3*** of dodecyl end-group). Mn,NMR = 5600 g mol^{-1} (DPPHEA, NMR = 44). SEC (5 mM NH4BF4 in DMF) Mn, SEC RI = 5600 g mol , $Đ$M, SEC RI = 1.27. FT-IR (neat): ν (cm) 3300 (N–H and O–H stretch); 2868 (alkyl C–H stretch); 1772 (C6F5C=O stretch); 1638(amide C=O stretch); 1544 (N–H bend); 1438 (alkane); 1216 (C–O stretch); 1060 (C–O stretch); 950 (C–F peak on shoulder of 1060 peak).

Functionalisation of PHEA with amino-glycans

As a representative example, PFP-PHEA (12 mg, 2.1 µmol), α-2,3'-SALac-t-NH_2 (**5**) (5.2 mg, 7.3 µmol, 3.5 eq) was dissolved in 1 mL DMF with 1 drop of TEA. The reaction was stirred at room temperature for 16 h. The polymer was precipitated into diethyl ether from methanol twice and dried under vacuum.

Functionalisation of PHEA with DBCO

PFP-PHEA (250 mg, 0.045 mmol), dibenzocylclooctyne-amine (40 mg, 0.14 mmol, 3 eq) were dissolved in 2 mL DMF. The reaction was stirred at room temperature for 16 h. The polymer was precipitated into diethyl ether from methanol twice and dried under vacuum. The resulting

polymer was an off white solid. IR indicated loss of C=O stretch corresponding to the PFP ester.

Capture of glycans onto DBCO-PHEA

In a typical reaction, DBCO-PHEA (1 mg, 0.18 μmol) and azidopropyl-linked glycan (3 eq) was dissolved in 1 mL milliQ water and left to react overnight on a tube roller. The solution was used immediately for immobilisation onto AuNPs.

Gold nanoparticle synthesis

55 nm gold nanoparticles were synthesised by a modified step growth method developed by Bastús *et al.*[7] A solution of 2.2 mM sodium citrate in Milli-Q water (150 mL) was heated under reflux for 15 min under vigorous stirring. After boiling had commenced, 1 mL of $HAuCl_4$ (25 mM) was injected. The colour of the solution changed from yellow to bluish gray and then to soft pink in 10 min, 1 mL was taken for DLS and UV/Vis analysis. Immediately after the synthesis of the Au seeds and in the same reaction vessel, the reaction was cooled until the temperature of the solution reached 90 °C. Then, 1 mL of a $HAuCl_4$ solution (25 mM) was injected. After 20 min, the reaction was finished. This process was repeated twice. After that, the sample was diluted by adding 85 mL of MilliQ water and 3.1 mL of 60 mM sodium citrate. This solution was then used as a seed solution, and three further portions of 1.6 mL of 25 mM $HAuCl_4$ were added with 20 min between each addition. Following completion of this step, 1 mL was taken for DLS and UV/Vis analysis. The sample was diluted by adding 135 mL of MilliQ water and 4.9 mL of 60 mM sodium citrate. This solution was then used as a seed solution, and the process was repeated with three further additions of 2.5 mL of 25 mM $HAuCl_4$, this solution was analysed by DLS and UV/Vis and target size of 35 nm was reached, and the solution was cooled. After that, the sample was diluted by adding 215 mL of MilliQ water and 7.8 mL of 60 mM sodium citrate. This solution was then used as a seed solution, and

three further portions of 3.9 mL of 25 mM $HAuCl_4$ were added with 20 min between each addition. Following completion of this step aliquots were taken for DLS and UV/Vis analysis. This solution was stored in the dark and used without further purification.

Gold nanoparticle functionalisation using glyco-PHEA polymers

100 μL of 1 mg.mL^{-1} of polymer solution was added to 1 mL of OD 1 particles and left for 30 minutes at room temperature on a tube roller. After 30 mins, particles were centrifuged at 7000 rpm the supernatant was removed and resuspended in 1 mL milliQ H_2O. This was repeated a further two times to ensure complete removal of any unattached polymer. Stability was confirmed by incubating in 10 mM HEPES buffer for 30 mins.

Lectin and Viral Binding Studies

Live attenuated influenza vaccine (LAIV) virus stocks

The five 6:2 re-assortant LAIV viruses used in this study were generated using an 8-plasmid reverse genetics system as described previously[8–11] with the HA and NA segments from egg-adapted human isolated wildtype (V1) viruses and six internal gene segments (PB2, PB1, PA, NP, M and NS) from the A/Ann Arbor/6/1960 master donor virus A (MDVA)[10]. Alternatively, HA proteins contained naturally occurring or engineered sequence changes relative to V1, referred to as either V8 or V11. Rescued LAIV viruses were inoculated in 10–11 day-old embryonated hen's eggs and harvested 72 hours post infection. Thereafter the pooled egg harvest was further clarified, centrifuged and concentrated to produce the viral stocks. The fluorescent focus assay (FFA) was used as the measurement of viral titre for the LAIV viruses with a strain-specific antibody, as previously described.[11]

Table S1: Fluorescent focus assay viral titres for the live attenuated influenza vaccine viruses used in this study.

LAIV Virus	FFA Titre (log10FFU/mL)
A/Bolivia/559/2013_V8	8.95
A/Hawaii/66/2019_V1	9.58
A/New Caledonia/20/1999_V1	9.53
A/Slovenia/2903/2015_V8	9.22
A/Darwin/6/2018_V11	9.07

Lectin-induced aggregation studies by Absorbance

A 2 mg.mL^{-1} stock solution of the lectin (MAL I or SNA) was made up in 10 mM HEPES buffer with 0.15 M NaCl, 0.1 mM $CaCl_2$ and 0.01 mM $MnCl_2$. 25 µL serial dilution was made up in the same buffer in a clear, flat bottom, half-area 96-well microtitre plate. 25 µL of the glycoAuNP were added to each well and incubated at room temperature for 30 mins. After 30 minutes, an absorbance spectrum was recorded from 450 nm -700 nm with 10 nm intervals.

LAIV virus-induced aggregation studies by Absorbance

LAIV virus titres were determined previously with FFA. Titres ranged from 8.95-9.58 log_{10} FFU/mL. 25 µL serial dilution of LAIV was made up in 10 mM HEPES 10 mM HEPES (pH 7.4), 150 mM NaCl, 0.005 % Tween 20, 4 mM $CaCl_2$, 25 µM oseltamivir carboxylate and 100 µM zanamivir, flat bottom, half-area 96-well microtitre plate. 25 µL of the glycoAuNP were added to each well and incubated at room temperature for 30 mins. After 30 minutes, an absorbance spectrum was recorded from 450 nm -700 nm with 10 nm intervals. Absorbance at 700 nm was plotted against dilution of LAIV. N.B. LAIVs were not cultured here, just diluted and incubated with the AuNPs. LAIVs were handled under Biosafety Level 2 containment conditions by trained personnel.

Biolayer interferometry (BLI)

BLI was carried out on ForteBio Octet Red96 (ForteBio, USA). Assays were performed in black, flat bottom, 96-well plates. Assays were carried out at 30 °C and agitated at 1000 rpm. Amine reactive biosensor tips (ARG2, Sartorius) were hydrated in milliQ H_2O water for at least 10 min prior to use. A stable baseline was established in milliQ water for 1 min. The biosensors were activated using 20 mM EDC and 40 mM sulfo-NHS for 5 min. Sensors were loaded with 75 µL LAIV in 125 µL pH 5 10 mM acetate buffer 10 min. Unreacted sites on the sensors were quenched using 100 mM ethanolamine for 10 mins followed by a 1 min equilibration step in 10 mM HEPES with 0.15 M NaCl and 0.1 mM CaCl2 and MnCl2 to remove any unbound LAIV and ethanolamine and to establish a stable baseline. Following virus immobilisation, the binding association with OD 1 glycoAuNPs was carried out in 10 mM HEPES 10 mM HEPES (pH 7.4), 150 mM NaCl, 0.005 % Tween 20, 4 mM $CaCl_2$, 25 µM oseltamivir carboxylate and 100 µM zanamivir for 30 min followed by dissociation in the same buffer for 10 min.

BLI loading optimisation

pH of the LAIV loading was first optimised (Figure S1).

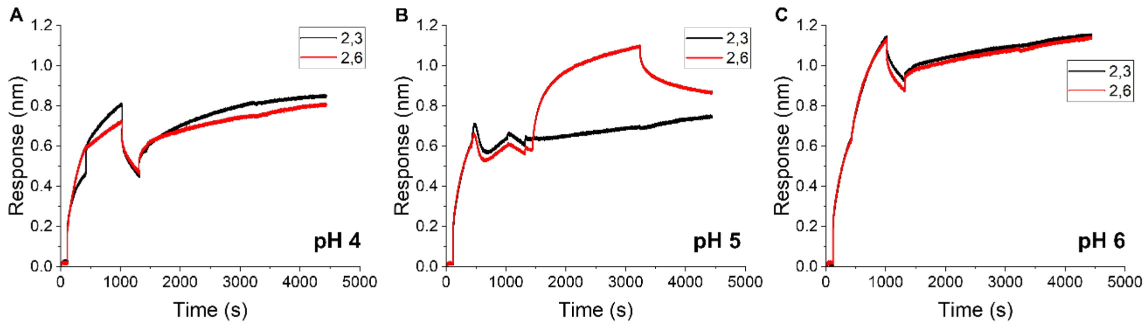

Figure S1: LAIV loading (0.5 × dilution) of A/Bolivia/599/2018_V8. at A) pH 4, B) pH 5 and C) pH 6 followed by association of 2,3 SL@AuNPs (black) and 2,6 SL@AuNPs (red).

pH 5 was found to be optimal for the loading of the LAIVs as gave as response. Following this, the LAIV loading concentration was determined.

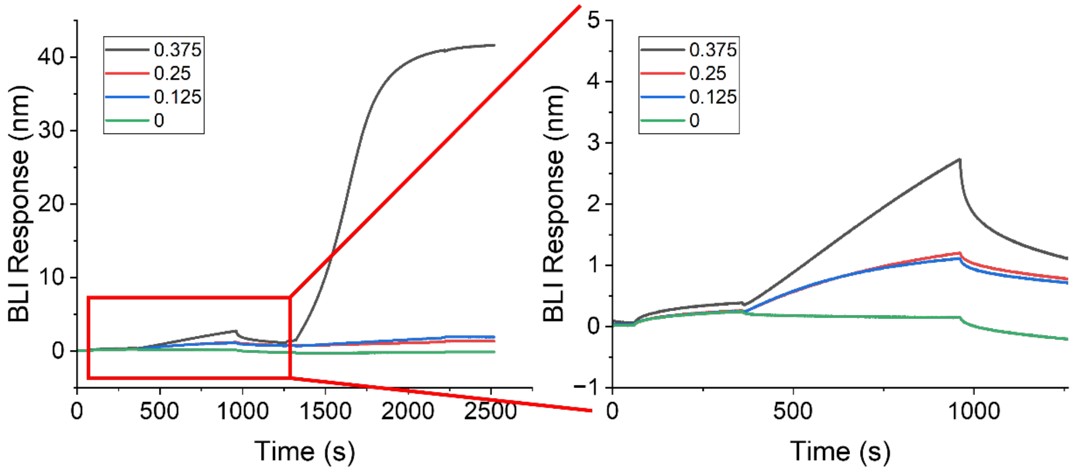

Figure S2: LAIV loading 0.3125, 0.25, 0,125 and 0 × dilution of A/Hawaii/66/2019_V1. Right hand side is a zoom of activation and loading steps.

A dilution of 0.375 × was found to give the highest response and therefore used going forward.

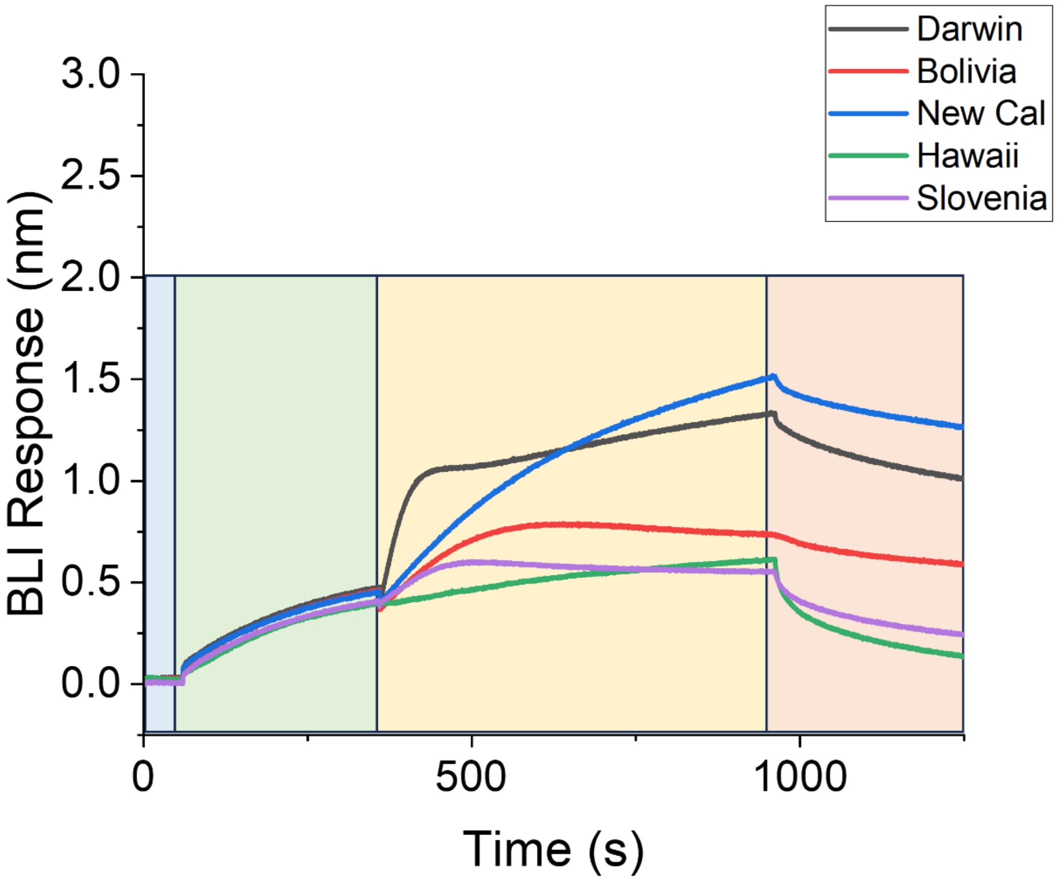

Figure S3: Comparison of the loading of each of the LAIVs used in this study.

Statistical Analysis

UV-Vis spectra were normalised by dividing through by the value at 450 nm. The error bars for binding isotherms are the standard error (Standard deviation/√n) where n is at least three measurements. No statistical analysis was carried out.

Nanoparticle Characterisation

Table S2: Characterisation of unfunctionalised and functionalised AuNPs used in this study.

Code	Glycan	UVmax[(a)] (nm)	Dh[(c)] (nm)	$D_{h\ (DLS)}$[(d)] (nm)
$AuNP_{60}$	-	536	60	57.8 ± 1.2
**5**-$PHEA_{44}$@$AuNP_{60}$	2,3SL	538	64	75.4 ± 6.4
**6**-$PHEA_{25}$@$AuNP_{60}$	2,6SL	540	68	75.8 ± 4.9
**17**-$PHEA_{25}$@$AuNP_{60}$	2,3SL/Lac	539	66	74.6 ± 4.5
**3**-$PHEA_{25}$@$AuNP_{60}$	Bis2,3SL	540	68	78.0 ± 5.2
**2**-$PHEA_{25}$@$AuNP_{60}$	Bis2,6SL	540	68	76.3 ± 3.5
**1**-$PHEA_{25}$@$AuNP_{60}$	Bis2,6SL Asym	541	70	88.5 ± 5.3
**16**-$PHEA_{25}$@$AuNP_{60}$	BisLac	540	68	78.0 ± 3.7

(a) SPR absorption maximum; (b) Absorbance ratio of SPR to 450 nm; (c) Estimated from UV-Vis[12]; (d) From dynamic light scattering.

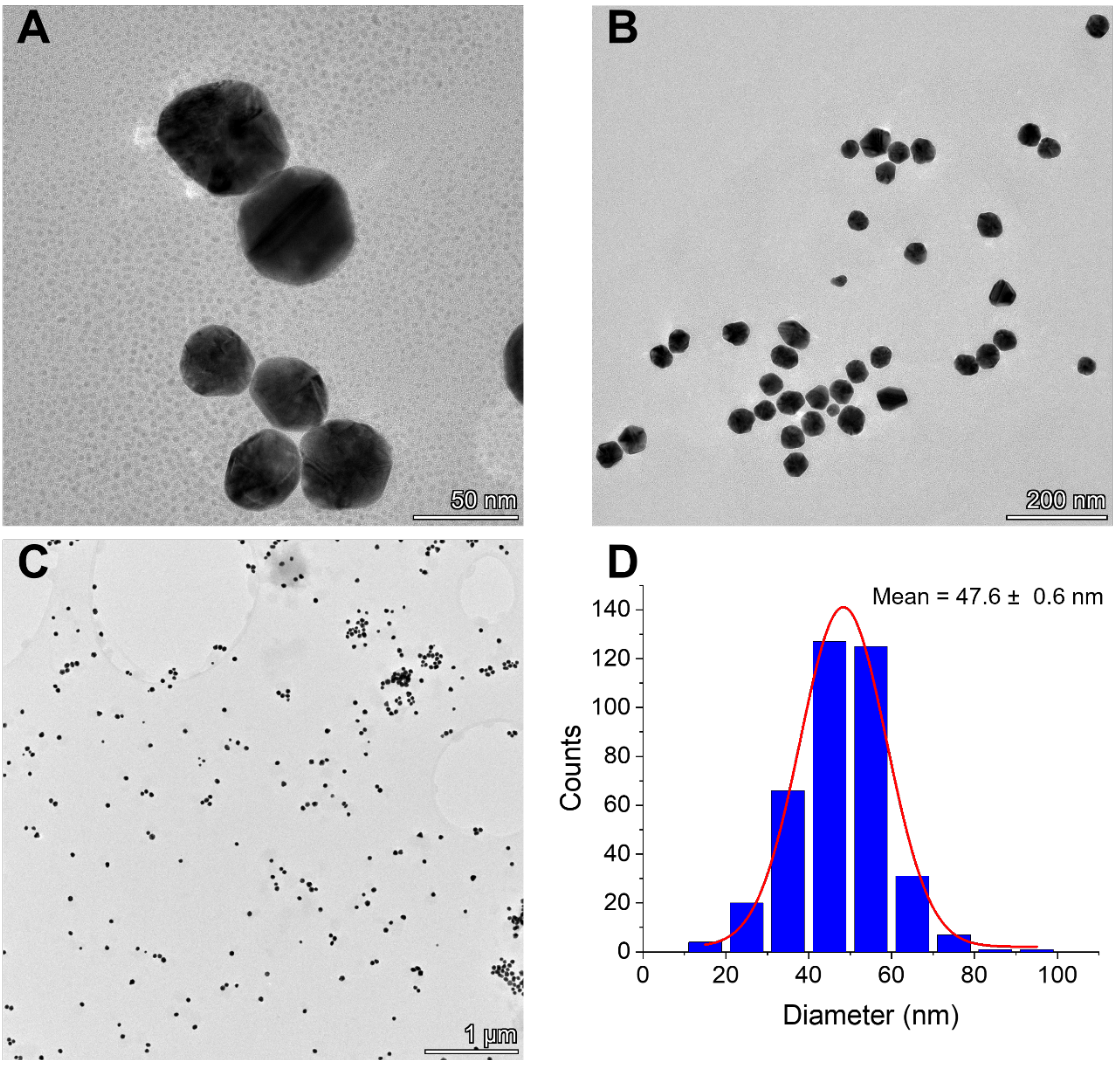

Figure S4: A), B) and C) TEMs of unfunctionalised AuNPs. D) Histogram of sizes from TEM analysis from over 300 particle measurements.

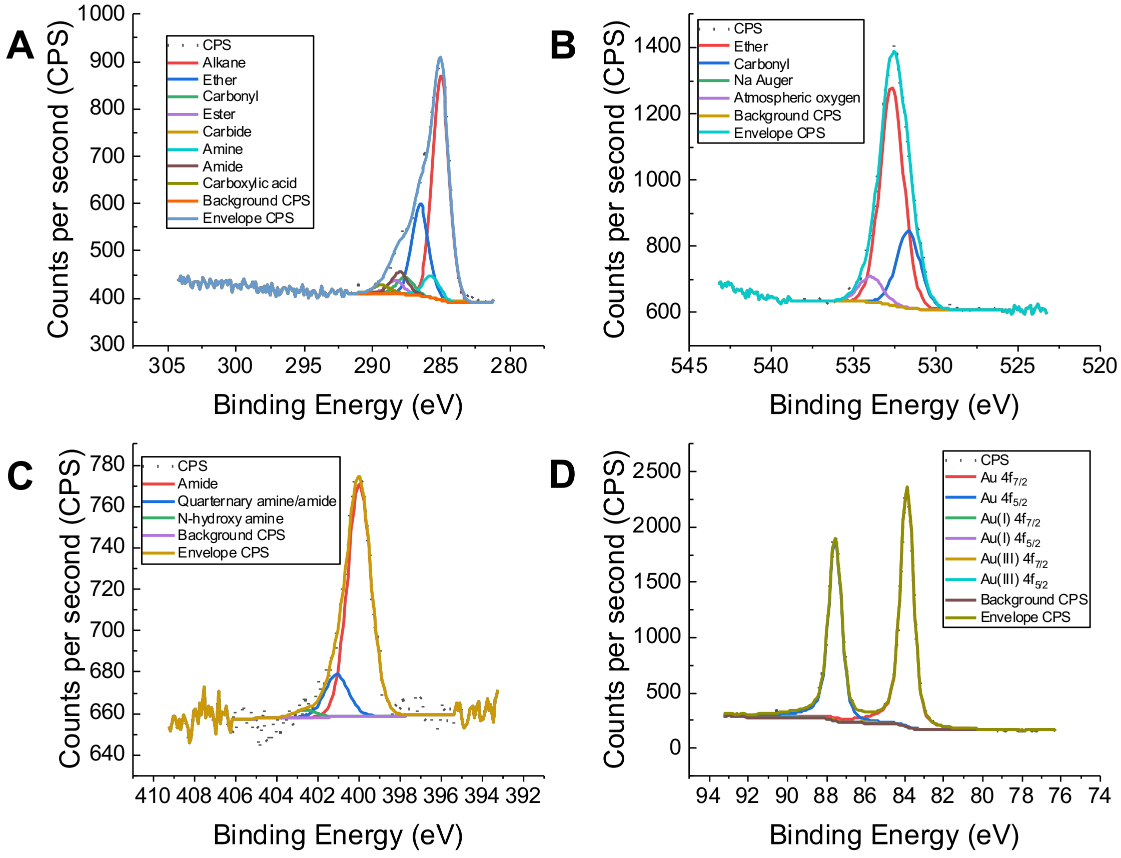

Figure S5: XPS of 2,3 SL-**5**-PHEA$_{44}$@AuNP$_{60}$ A) C 1s B) O 1s C) N 1s and D) Au 4f

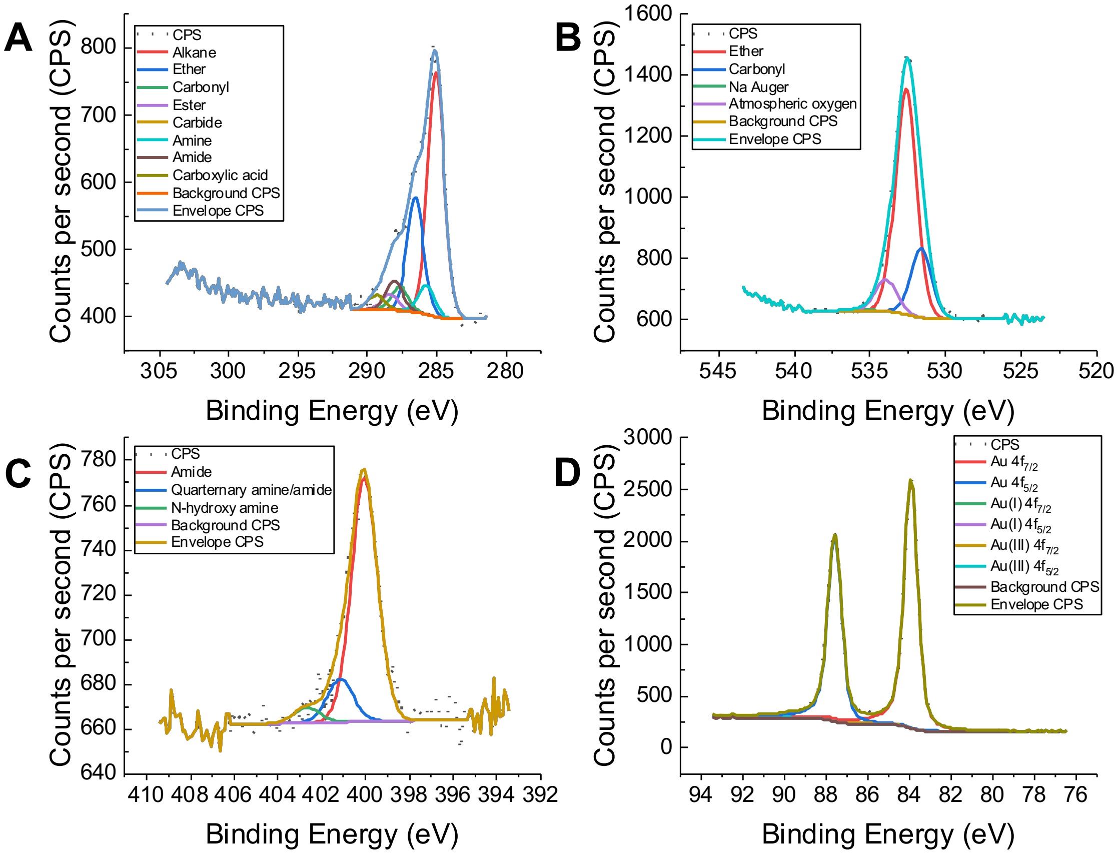

Figure S6: XPS of 2,6 SL-**6**-$PHEA_{44}$@$AuNP_{60}$ A) C 1s B) O 1s C) N 1s and D) Au 4f

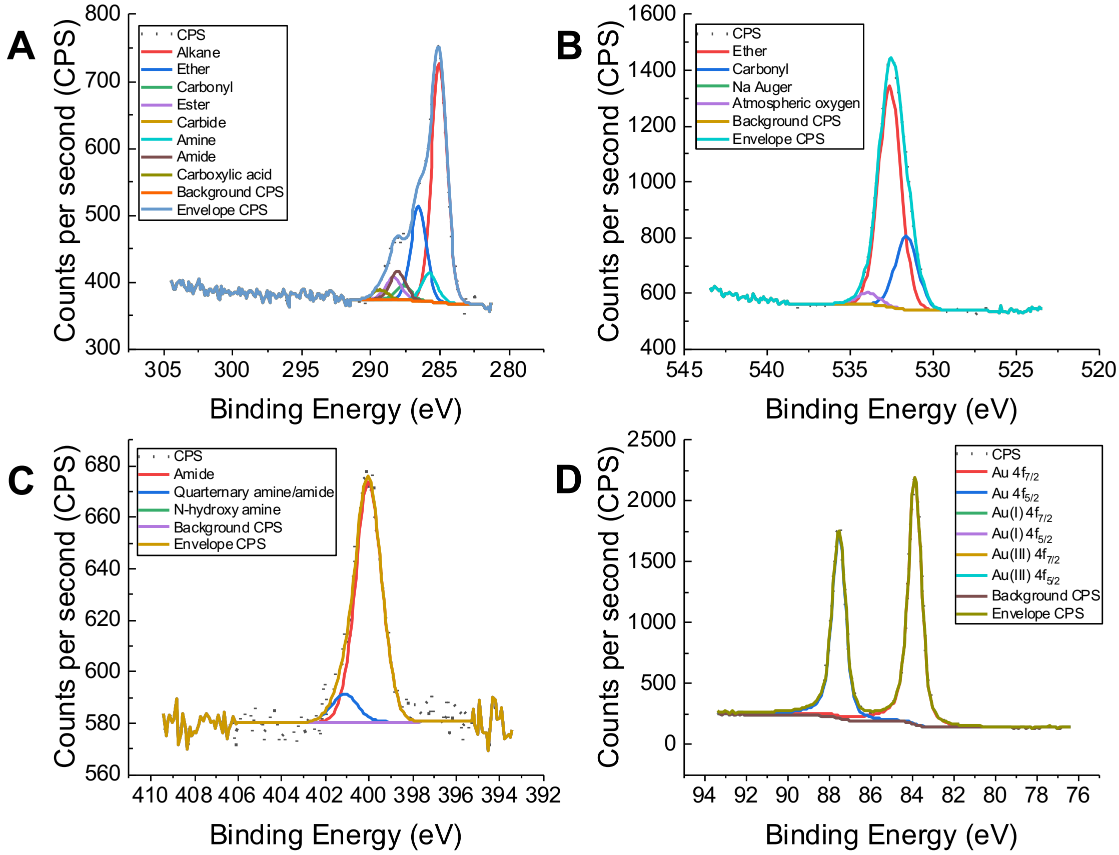

Figure S7: XPS of Bis2,6 SL-**2**-PHEA$_{44}$@AuNP$_{60}$ A) C 1s B) O 1s C) N 1s and D) Au 4f

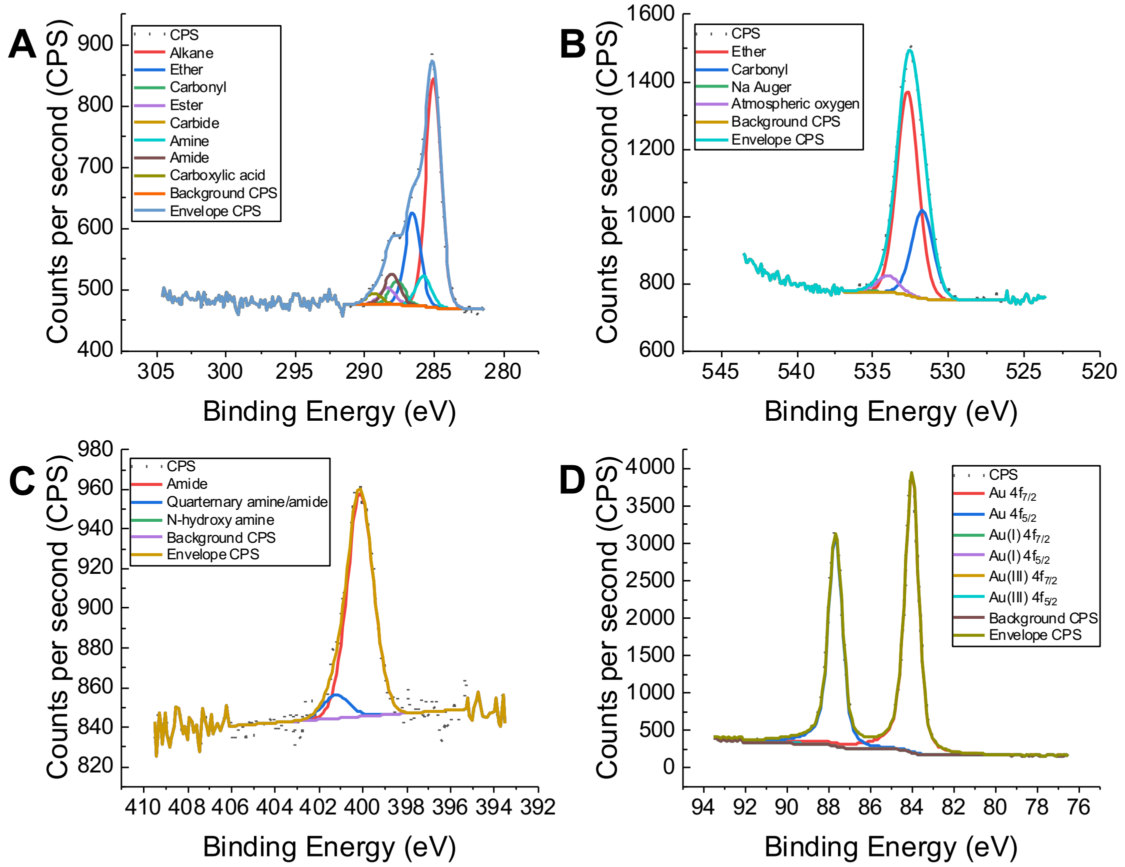

Figure S8: XPS of Bis2,6 SL Asym-**1**-PHEA$_{44}$@AuNP$_{60}$ A) C 1s B) O 1s C) N 1s and D) Au 4f.

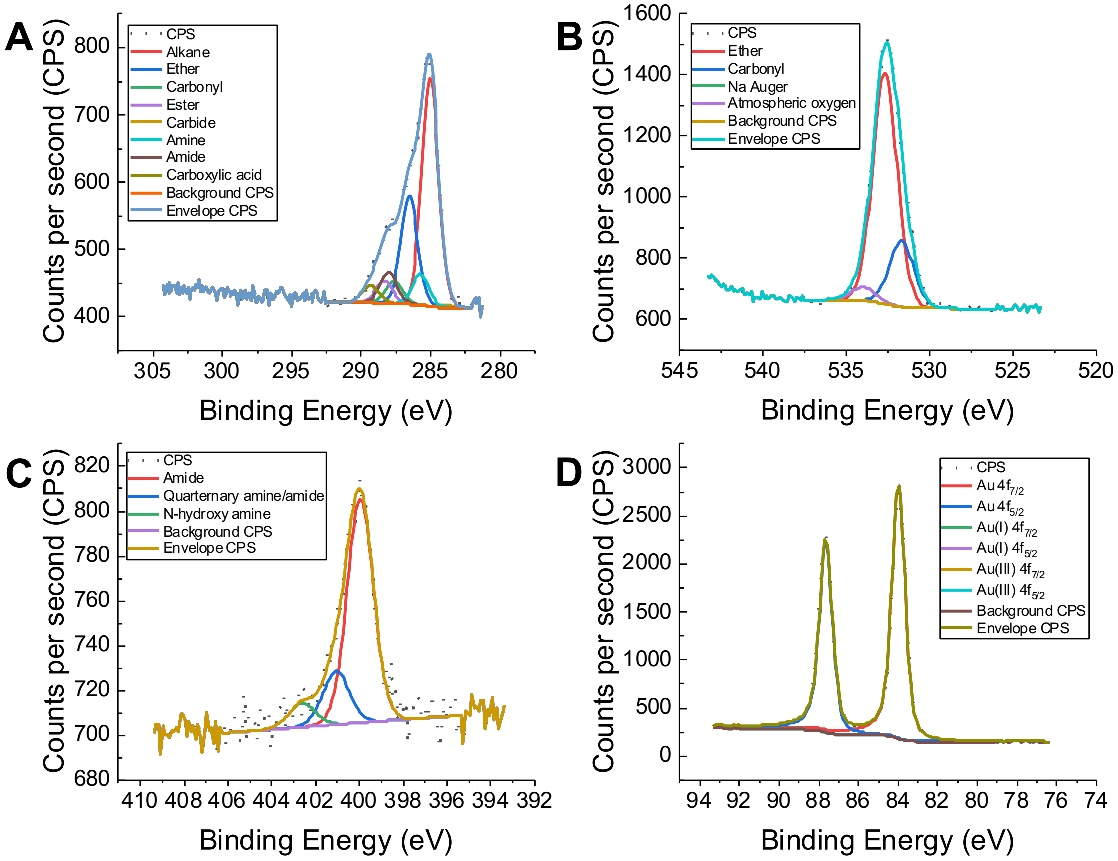

Figure S9: XPS of BisLac-**16**-PHEA$_{44}$@AuNP$_{60}$ A) C 1s B) O 1s C) N 1s and D) Au 4f

Kinetic of colour change of AuNP in response to LAIVs using UV-Vis

Absorbance at 700 nm over time of 2,3SL-PHEA@AuNP and 2,6-PHEA@AuNP in response to 0.01 x dilution of A/Hawaii/66/2019_V1

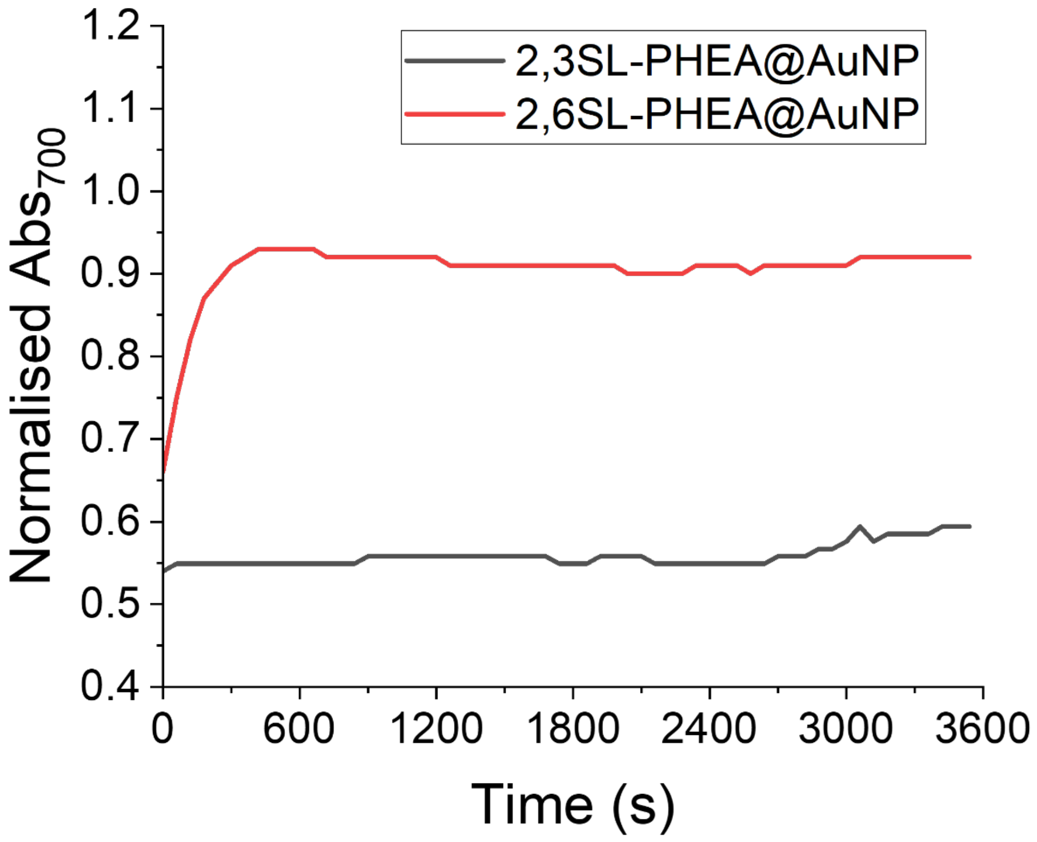

Figure S10: Absorbance of at 700 nm normalised to the absorbance at 450 nm taken every 30 s for 1 hour of 2,3SL-PHEA@AuNP (black) and 2,6SL-PHEA@AuNP (red) in response to 0.01 x dilution of A/Hawaii/66/2019_V1.

This shows that the colour change occurs quickly within the first 10 mins and is stable over at least 1 hour. Subsequent measurements were taken after 30 mins.

Absorbance spectra of AuNPs in response to LAIVs

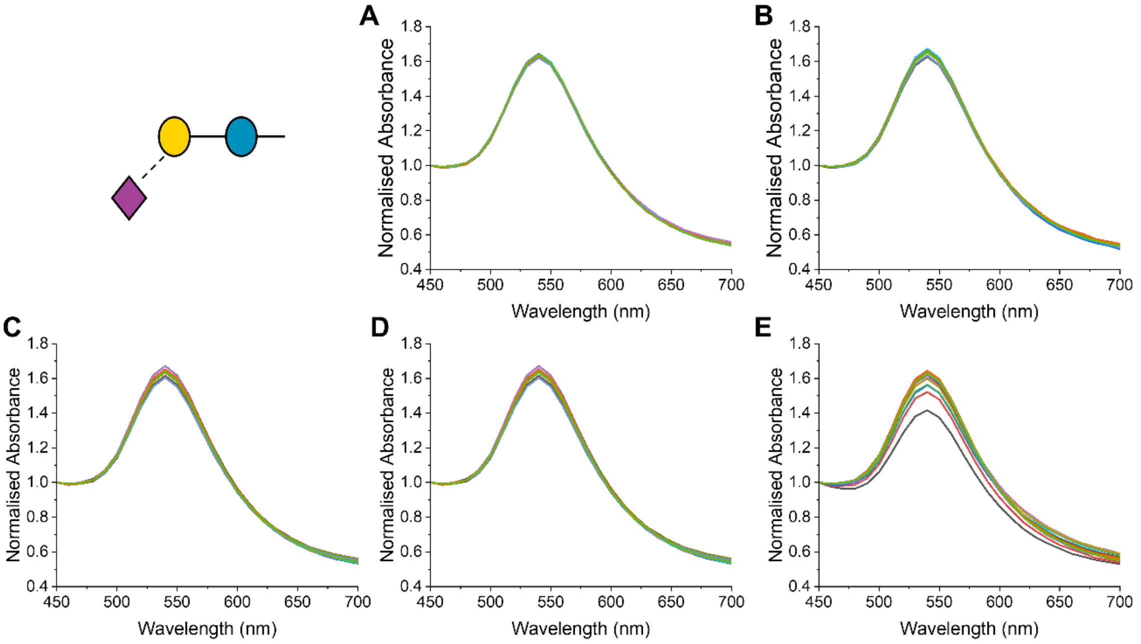

Figure S11: Absorbance spectra of 2,3SL-**5**-PHEA$_{44}$@AuNP$_{60}$ in response to a serial dilution of A) A/Hawaii/66/2019_V1 B) A/New Caledonia/20/1999/V1 C) A/Bolivia/559/2018_V8 D) A/Slovenia/2903/2015/V8 and E) A/Darwin/6/2018_V11.

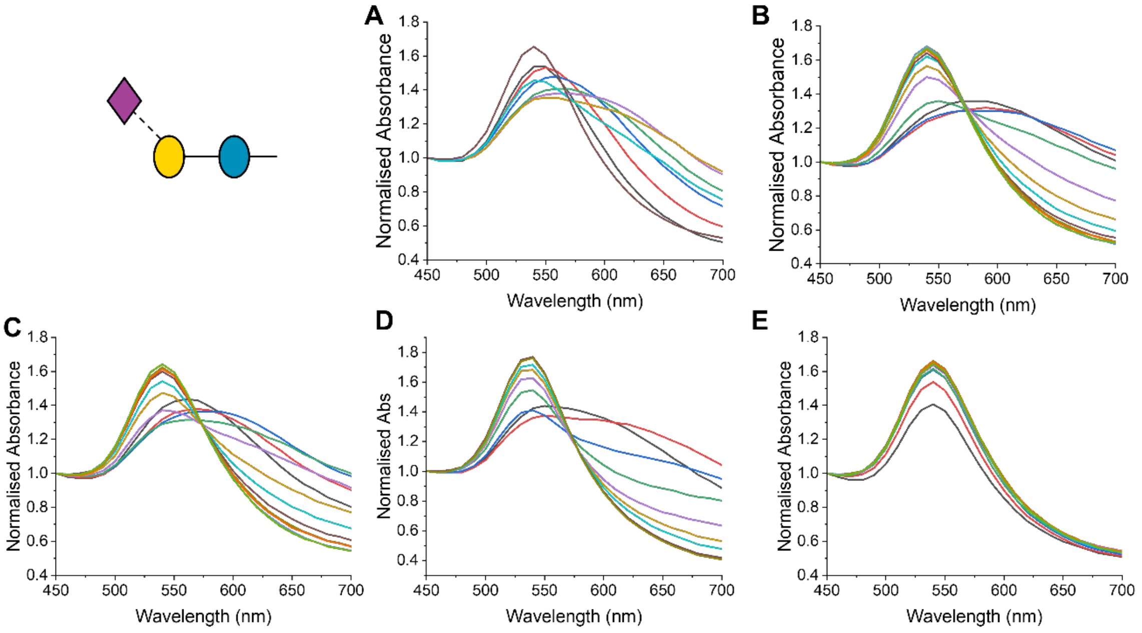

Figure S12: Absorbance spectra of 2,6SL-**6**-PHEA$_{44}$@AuNP$_{60}$ in response to a serial dilution of A) A/Hawaii/66/2019_V1 B) A/New Caledonia/20/1999/V1 C) A/Bolivia/559/2018_V8 D) A/Slovenia/2903/2015/V8 and E) A/Darwin/6/2018_V11.

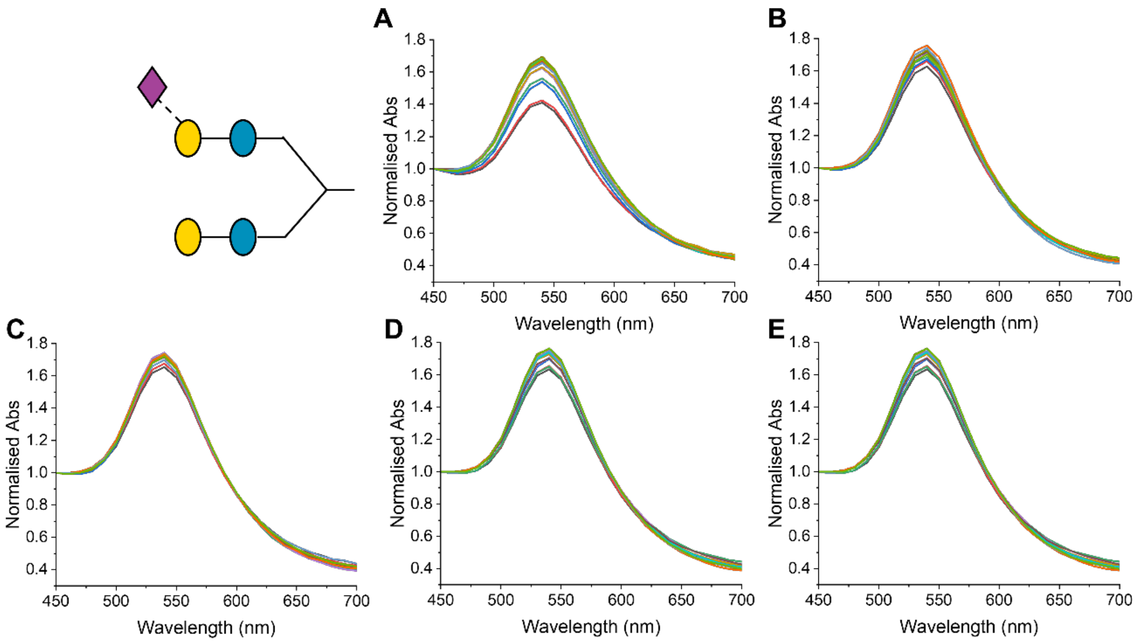

Figure S13: Absorbance spectra of 2,3SL/Lac-**17**-PHEA$_{44}$@AuNP$_{60}$ in response to a serial dilution of A) A/Hawaii/66/2019_V1 B) A/New Caledonia/20/1999/V1 C) A/Bolivia/559/2018_V8 D) A/Slovenia/2903/2015/V8 and E) A/Darwin/6/2018_V11.

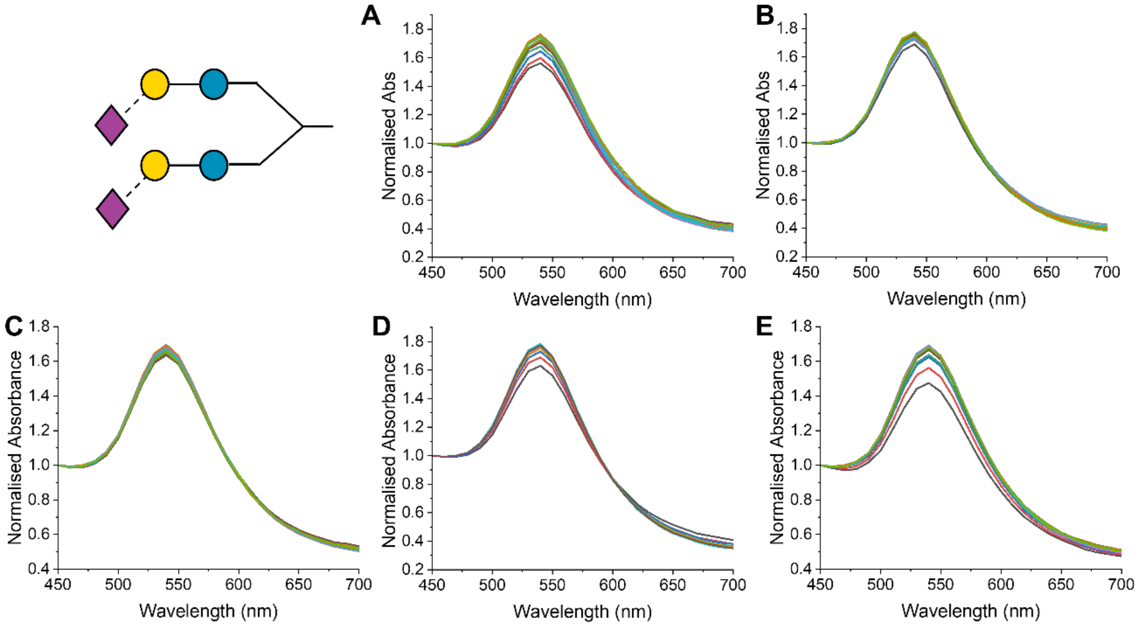

Figure S14: Absorbance spectra of bis2,3SL-**3**-PHEA$_{44}$@AuNP$_{60}$ in response to a serial dilution of A) A/Hawaii/66/2019_V1 B) A/New Caledonia/20/1999/V1 C) A/Bolivia/559/2018_V8 D) A/Slovenia/2903/2015/V8 and E) A/Darwin/6/2018_V11.

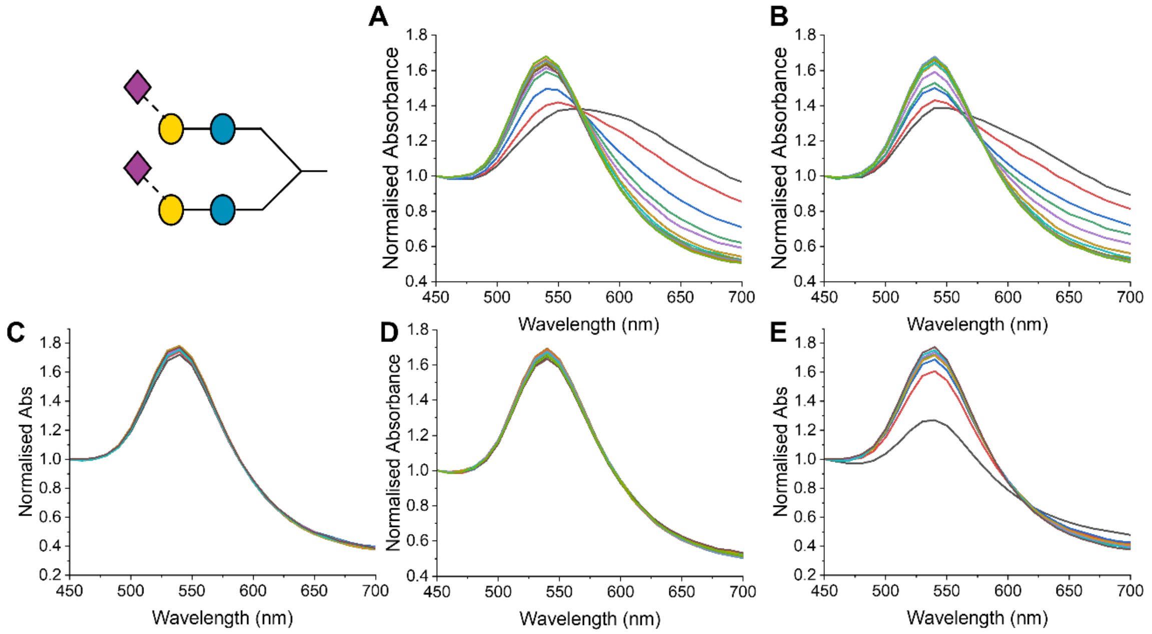

Figure S15: Absorbance spectra of bis2,6SL-**2**-PHEA$_{44}$@AuNP$_{60}$ in response to a serial dilution of A) A/Hawaii/66/2019_V1 B) A/New Caledonia/20/1999/V1 C) A/Bolivia/559/2018_V8 D) A/Slovenia/2903/2015/V8 and E) A/Darwin/6/2018_V11.

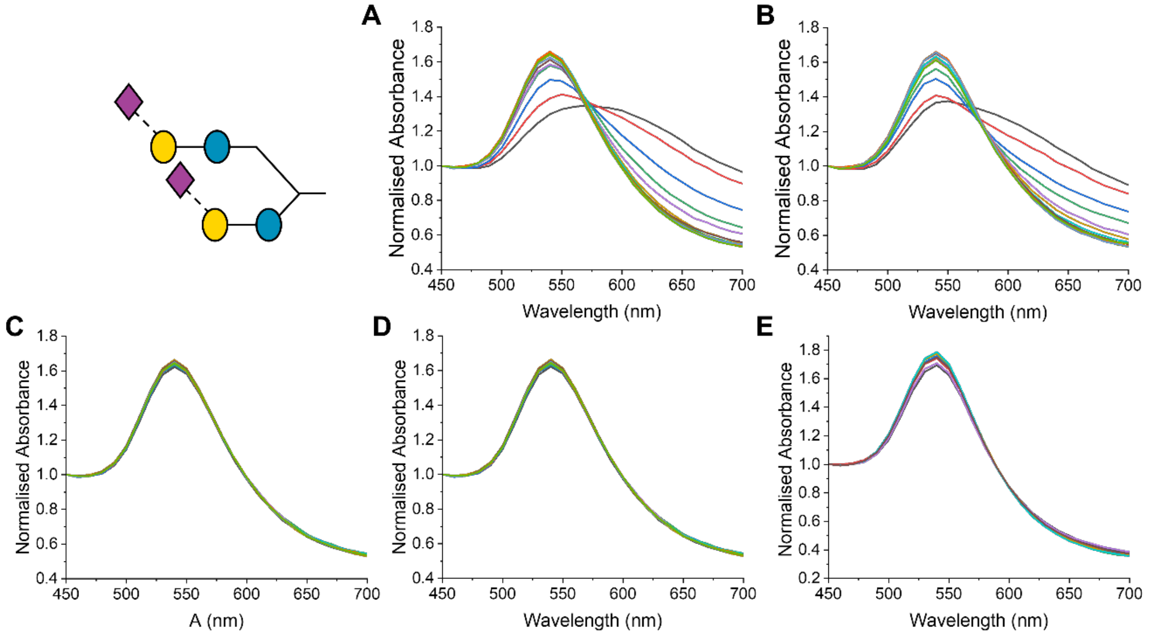

Figure S16: Absorbance spectra of Bis2,6SL Asym-**1**-PHEA$_{44}$@AuNP$_{60}$ in response to a serial dilution of A) A/Hawaii/66/2019_V1 B) A/New Caledonia/20/1999/V1 C) A/Bolivia/559/2018_V8 D) A/Slovenia/2903/2015/V8 and E) A/Darwin/6/2018_V11.

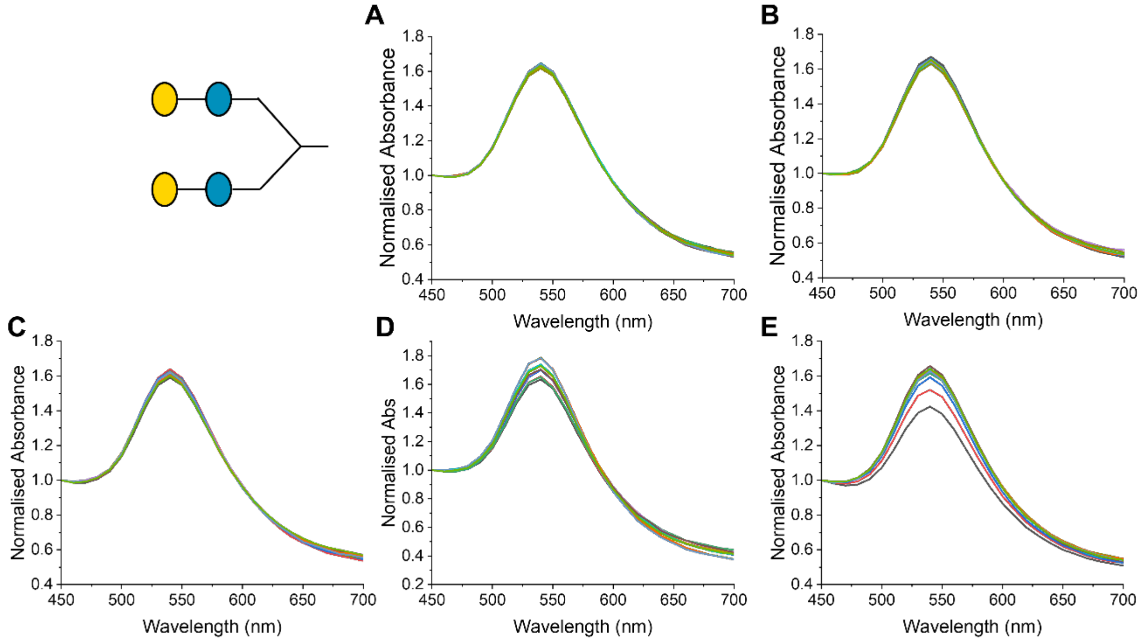

Figure S17: Absorbance spectra of of **16**-$PHEA_{25}$@$AuNP_{60}$ in response to a serial dilution of A) A/Hawaii/66/2019_V1 B) A/New Caledonia/20/1999/V1 C) A/Bolivia/559/2018_V8 D) A/Slovenia/2903/2015/V8 and E) A/Darwin/6/2018_V11.

References

(1) Dedola, S.; Hughes, D. L.; Nepogodiev, S. A.; Rejzek, M.; Field, R. A. Synthesis of α- and β-d-Glucopyranosyl Triazoles by CuAAC "Click Chemistry": Reactant Tolerance, Reaction Rate, Product Structure and Glucosidase Inhibitory Properties. *Carbohydr. Res.* **2010**, *345*, 1123–1134.

(2) Gao, Y.; Chen, L.; Zhang, Z.; Gu, W.; Li, Y. Linear Cationic Click Polymer for Gene Delivery: Synthesis, Biocompatibility, and in Vitro Transfection. *Biomacromolecules* **2010**, *11*, 3102–3111.

(3) Fairbanks, A. J. Applications of Shoda's Reagent (DMC) and Analogues for Activation of the Anomeric Centre of Unprotected Carbohydrates. *Carbohydr. Res.* **2021**, *499*, 108197.

(4) Akula, R. A.; Temelkoff, D. P.; Artis, N. D.; Norris, P. Rapid Access to Glucopyranosyl-1,2,3-triazoles via Cu(I)-Catalyzed Reactions in Water. *ChemInform* **2005**, *36*, no-no.

(5) Campo, V. L.; Carvalho, I.; Allman, S.; Davis, B. G.; Field, R. A. Chemical and Chemoenzymatic Synthesis of Glycosyl-Amino Acids and Glycopeptides Related to Trypanosoma Cruzi Mucins. *Org. Biomol. Chem.* **2007**, *5*, 2645–2657.

(6) Campo, V. L.; Carvalho, I.; Da Silva, C. H. T. P.; Schenkman, S.; Hill, L.; Nepogodieva, S. A.; Field, R. A. Cyclooligomerisation of Azido-Alkyne-Functionalised Sugars: Synthesis of 1,6-Linked Cyclic Pseudo-Galactooligosaccharides and Assessment of Their Sialylation by Trypanosoma Cruzi Trans-Sialidase. *Chem. Sci.* **2010**, *1*, 507–514.

(7) Bastús, N. G.; Comenge, J.; Puntes, V. Kinetically Controlled Seeded Growth Synthesis of Citrate-Stabilized Gold Nanoparticles of up to 200 Nm: Size Focusing versus Ostwald Ripening. *Langmuir* **2011**, *27*, 11098–11105.

(8) Hoffmann, E.; Neumann, G.; Kawaoka, Y.; Hobom, G.; Webster, R. G. A DNA Transfection System for Generation of Influenza A Virus from Eight Plasmids. *Proc. Natl. Acad. Sci. U. S. A.* **2000**, *97*, 6108–6113.

(9) Chen, Z.; Wang, W.; Zhou, H.; Suguitan, A. L.; Shambaugh, C.; Kim, L.; Zhao, J.; Kemble, G.; Jin, H. Generation of Live Attenuated Novel Influenza Virus A/California/7/09 (H1N1) Vaccines with High Yield in Embryonated Chicken Eggs. *J. Virol.* **2010**, *84*, 44–51.

(10) Chan, W.; Zhou, H.; Kemble, G.; Jin, H. The Cold Adapted and Temperature Sensitive Influenza A/Ann Arbor/6/60 Virus, the Master Donor Virus for Live Attenuated Influenza Vaccines, Has Multiple Defects in Replication at the Restrictive Temperature. *Virology* **2008**, *380*, 304–311.

(11) Dibben, O.; Crowe, J.; Cooper, S.; Hill, L.; Schewe, K. E.; Bright, H. Defining the Root Cause of Reduced H1N1 Live Attenuated Influenza Vaccine Effectiveness: Low Viral Fitness Leads to Inter-Strain Competition. *npj Vaccines* **2021**, *6*, 35.

(12) Haiss, W.; Thanh, N. T. K.; Aveyard, J.; Fernig, D. G. Determination of Size and Concentration of Gold Nanoparticles from UV - Vis Spectra. *Anal. Chem.* **2007**, *79*, 4215–4221.
